# Diethyldithiocarbamate-copper complex ignites the tumor microenvironment through NKG2D-NKG2DL axis

**DOI:** 10.3389/fimmu.2025.1491450

**Published:** 2025-02-12

**Authors:** Daciana C. Dumut, Marian Hajduch, Amanda M. Zacharias, Qingling Duan, Ivo Frydrych, Zuzana Rozankova, Miroslav Popper, Dusan Garic, Radu Alexandru Paun, Amanda Centorame, Juhi Shah, Martin Mistrik, Petr Dzubak, Juan B. De Sanctis, Danuta Radzioch

**Affiliations:** ^1^ Department of Experimental Medicine, Faculty of Medicine, McGill University, Montreal, QC, Canada; ^2^ The Research Institute of the McGill University Health Centre, Infectious Diseases in Global Health Program, Montreal, QC, Canada; ^3^ Institute of Molecular and Translational Medicine, Faculty of Medicine and Dentistry, Palacky University, Olomouc, Czechia; ^4^ Czech Advanced Technology and Research Institute, Palacky University Olomouc, Olomouc, Czechia; ^5^ Department of Biomedical & Molecular Sciences, Faculty of Health Sciences, Queen’s University, Kingston, ON, Canada; ^6^ School of Computing, Department of Biomedical and Molecular Sciences, Queen’s University, Kingston, ON, Canada; ^7^ Department of Developmental Neurobiology, St. Jude Children’s Research Hospital, Memphis, TN, United States; ^8^ Department of Biomedical Engineering, McGill University, Montreal, QC, Canada

**Keywords:** copper bis-diethyldithiocarbamate, disulfiram, colorectal cancer, NK cells, NKG2D

## Abstract

Advanced metastatic colorectal cancer (CRC) with deficient DNA mismatch repair (MMR-d), or immune-hot CRCs, show significantly improved clinical outcomes compared to MMR-proficient (MMR-p), or immune-cold CRCs. While the prior represents about 5% of all CRCs, the latter represent 95% and are characterized by low immunogenicity. This study investigates bis-diethyldithiocarbamate (CuET), a novel anticancer compound, and its impact on the colorectal cancer tumor microenvironment (TME). CuET is shown to convert immunologically inactive tumors into hotbeds of antitumor immune responses, marked by increased lymphocyte infiltration, heightened cytotoxicity of natural killer (NK) and T cells, and enhanced non-self recognition by lymphocytes. The potent anticancer cytotoxicity and *in vivo* safety and efficacy of CuET are established. In summary, CuET transforms the colorectal cancer TME, bolstering NK and T cell cytotoxicity and refining tumor cell recognition through non-classical activation via the NKG2D/NKG2DL axis. This study unveils a novel mechanism of action for CuET: a potent immunomodulator capable of turning cold tumors hot.

## Graphical Abstract

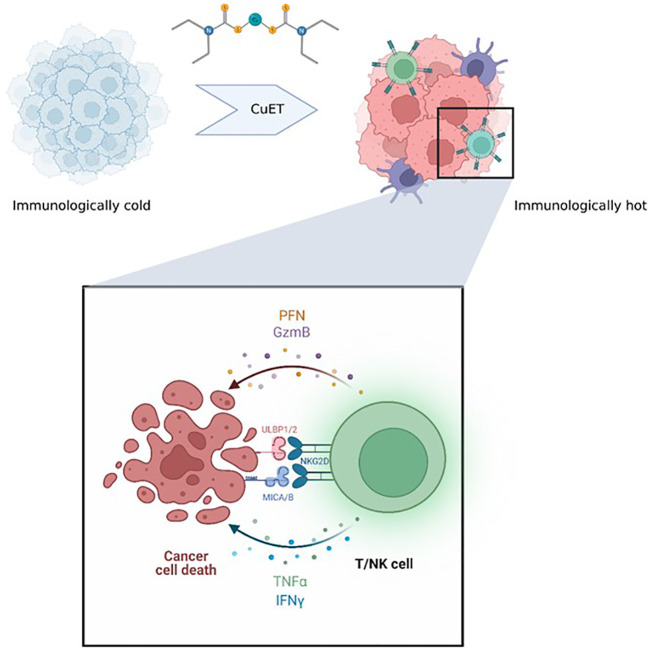

## 1 Introduction

Colorectal cancer (CRC) ranks second in cancer-related deaths globally and is primarily associated with lifestyle. While surgical resection and chemotherapy improve survival in localized disease, distant-stage disease or metastatic CRC (mCRC) has a poor prognosis, with a projected 5-year survival rate of only 14% (1). CRC pathogenesis begins through polyp development, progressing to cancer over an estimated 10-15 years due to genetic and epigenetic alterations inactivating tumor suppressor genes (TP53, APC, MADR2, MUTYH, STK11, SMAD2/4, etc.) and activating oncogenes (2). Dysfunctional DNA mismatch repair (MMR) mechanisms, leading to microsatellite instability (MSI), also contribute to CRC etiology (2).

The mutational landscape, although insufficient for sub-classifying tumor types or predicting patient survival in colorectal cancer (CRC), significantly influences the response to immunotherapy. A Phase 2 clinical study (NCT01876511) found that patients with MMR-deficient (MMR-d) advanced metastatic CRC benefited more from pembrolizumab immune checkpoint inhibition compared to those with MMR-proficient (MMR-p) CRCs (3). This difference was attributed to the higher lymphocyte infiltration observed in MSI/MMR-d tumors, marked by increased somatic mutations and non-self neoantigen presentation, compared to MSS/MMR-p tumors. While only approximately 5% of CRCs, characterized by MMR-d, respond well to immunotherapy, the remaining 95%, characterized by MMR-p and low tumor immunogenicity, exhibit reduced responsiveness (4).

Enhancing lymphocyte recruitment into immunologically inert “cold” tumors is crucial for creating an immune-responsive milieu, as underscored by studies emphasizing the critical role of the tumor microenvironment (TME) and lymphocyte infiltration as prognostic indicators for cancer treatment (5, 6). Increased somatic mutation burden and neoantigen production are pivotal for enhancing immunogenicity, promoting robust non-self recognition by lymphocytes, and eliciting a potent antitumor response (7). Thus, improving immunotherapy response rates in CRC hinges on enhancing neoantigen production, antigen recognition, and lymphocyte infiltration in non-hypermutated MSS/MMR-p tumors, promoting a shift from cold to hot tumor profiles. Adjuvant drugs induce higher tumor immunogenicity and lymphocyte responses, potentially increasing the response rates in refractory solid tumors (8).

Disulfiram (Antabuse, DSF), initially approved for alcoholism treatment in 1949, has recently gained attention in cancer research due to its antineoplastic activity in preclinical studies (9, 10). Indeed, multiple groups have reported on the efficacy of various DSF formulations such as nanoparticles, liposomes, copper complexes, and iron complexes in colorectal cancer preclinical models (11–15).

DSF is metabolized in the gastrointestinal tract and bloodstream, resulting in the production of diethyldithiocarbamate (DDTC), a potent chelator of bivalent metals like Cu^2+^ and Zn^2+^. Upon DDTC chelation of Cu^2+^, the primary anticancer agent, bis-diethyldithiocarbamate-copper (CuET), is formed. While DSF has demonstrated high and efficient cytotoxicity in pre-clinical studies, the clinical benefit, as shown by trials, is limited (16–18). It has been hypothesized that modest results in clinic can be attributed to DSF’s short half-life in the blood, of approximately 2-4 minutes, in its current oral formulation (19). Since the anticancer effects of DSF are primarily dependent on the formation of the active metabolite, CuET, direct administration of CuET is likely to be more effective (17, 20). Skrott et al. developed an albumin-based CuET formulation suitable for *in vivo* pre-clinical studies (21).

The cytotoxic effects of DSF are attributed to various mechanisms, including i) chelation of divalent cations (Cu2+ and Zn2+) by its metabolite DDTC (20), ii) NF-κB signaling inhibition (22), iii) MAPK pathway activation (23), iv) proteasomal degradation inhibition and induction of proteotoxic stress (24), v) oxidative stress via reactive oxygen species production (25), vi) heat-shock protein induction (26), vii) DNA methyltransferase inhibition (27), and viii) p97-NPL4 pathway immobilization (21). These actions disrupt critical cancer pathways, such as angiogenesis, hypoxia signaling, and P-glycoprotein pump resistance, leading to apoptosis and autophagy in cancer cells (28–30).

Additionally, the p97-NPL4 pathway is crucial for maintaining protein homeostasis by participating in the ubiquitin-proteasome system (UPS), which regulates protein degradation. Impairment of this pathway results in misfolded protein aggregation, DNA damage, reactive oxygen species (ROS) production, and enhanced immune activation. These effects facilitate the recognition of cancer cells by cytotoxic lymphocytes and more specifically the anticancer response (21, 31). Although the inhibition of aldehyde dehydrogenase (ALDH) has also been proposed as one of the anticancer mechanisms of DSF, evidence indicates that its anticancer effects are attributed to the targeting of NPL4 rather than ALDH (21).

Recent studies have highlighted the ability of DSF and DSF/Cu to enhance antitumor immunity by improving the efficacy of programmed cell death protein 1 (PD-1) therapy and as directly activating CD8+ T cells (32, 33). Voli et al. demonstrated that copper-chelating drugs increased the presence of tumor-infiltrating CD8+ T and NK cells, suggesting that reducing intratumoral copper levels could enhance anticancer immunotherapy efficacy (34). While the immunoadjuvant properties of CuET are not yet fully understood, evidence of its role in promoting T cell and NK cell cytotoxicity is steadily growing.

Natural killer group 2D (NKG2D) is a C-type lectin-like immune receptor expressed on all human NK, NKT, CD8+ T cells, and a subset of γδ T cells (35, 36). The human NKG2D receptor recognizes and binds to MHC class 1 chain-related molecules A and B (MICA, MICB) as well as to six cytomegalovirus UL16-binding proteins (ULBP1-ULBP6). Engagement of the NKG2D receptor by its ligands (NKG2DLs) provides an activating signal to NK cells and co-stimulates T cells (37).

While NKG2DLs are typically absent or expressed at very low levels on the surface of normal cells, they are often present on tumor cells, where their expression can be upregulated by radiation and chemotherapy to enhance NK cell-mediated anticancer cytotoxicity (38). Conversely, tumor cells can evade immune detection through mechanisms that involve the shedding of NKG2DLs, such as MICA/B. The shedding of NKG2DLs, mediated by tumor-secreted metalloproteases or the release of NKG2DLs via exosomes, leads to soluble MICA/B that can bind to NKG2D receptors thereby impairing the effector functions of NK and T cell that rely on NKG2D signaling (39). Goto et al. demonstrated that DSF inhibits a disintegrin and metalloproteinase 10 (ADAM10), leading to the upregulation of membrane-bound MICA ligands on hepatocellular carcinoma cells (40).

In this study, we hypothesize that CuET, the primary anticancer metabolite derived from DSF, may serve as an immunomodulator within the NKG2D-NKG2DL axis, enhancing the effector functions of NK, NKT, and T cell against CRC.

This study explores the ability of CuET to induce cytotoxic responses in various CRC cell lines and assesses the safety and efficacy of an albumin based CuET nanoparticle formulation in three murine CRC models, including one replicating metastasis. It establishes, for the first time, the dual role of CuET in stimulating NKG2D activating receptor expression in NK and T lymphocytes, and its effect on corresponding ligands in tumor cells, bolstering anticancer cytotoxicity. Transcriptomic analysis of NKG2D tumor ligands from The Cancer Genome Atlas (TCGA) underscore the importance of the NKG2D-NKG2DL axis in anticancer immunity. These findings reveal a novel mechanism by which CuET transforms the CRC microenvironment, turning cold tumors hot, through enhanced NK and T cell cytotoxicity and improved tumor cell recognition.

## 2 Methods

### 2.1 Cell culture

Murine CRC cell lines MC-38 (MMR-d, MSI) were kindly provided by Dr. Pnina Brodt, McGill University, who obtained them from Dr. Shoshana Yakar, New York University. CT-26 (MMR-p, MSS) were generously provided by Dr. Nicole Beauchemin of McGill University, sourced directly from Dr. Michael Brattain of the University of Nebraska Medical Centre. Dr. Brattain originally established the cell line in 1980 and granted permission to Dr. Beauchemin to share this cell line with other investigators (41). The human CRC cell lines HT-29 (MMR-p, MSS) (ATCC HTB-38) and HCT116 parental cells (KRAS^G13D^/KRAS^WT^, ATCC CCL-247, Horizon Cat. HD PAR-007) and HCT116 WT (KRAS ^WT^/KRAS ^KO^, cat. HD-104-008), and HCT116 KRAS^G13D^ (KRAS^G13D^/KRAS ^KO^, cat. HD 104-011) (MMR-d) were obtained from Horizon Discovery, Ltd. Growth conditions can be found in the **Supplementary Material**.

### 2.2 Cell proliferation and cytotoxicity assay

Cytotoxicity was measured using the sulforhodamine B assay as previously described (42).

Cells were detached with 0.05% trypsin-EDTA and seeded at 3 × 10^3^ cells per well in 96-well plates. 24h later, the cells were treated with serial dilutions of copper (II) diethyldithiocarbamate (TCI Chemicals) reconstituted in DMSO (Sigma). DMSO concentration in cell suspension was normalized across all wells and did not exceed 0.1%. Cells were incubated at 37°C, 5% CO_2_ for 72h. Cultures were fixed with 50% (w/v) trichloroacetic acid (Sigma) and stained for 30 min with 0.4% (w/v) sulforhodamine B (SRB; Alfa Aesar) dissolved in 1% (w/v) acetic acid (Sigma). Unbound dye was removed through four washes with 1% acetic acid solution, and protein-bound dye was extracted using 10 mM unbuffered Tris base (FisherScientific). OD492nm was measured. IC50 values were determined by calculating the percentage of cells killed in each test well as compared to the DMSO control well using the formula: % cells killed = 100 – (mean OD sample)/(mean OD control) x 100. IC50 values were determined by plotting a dose–response curve between the compound concentration and percent growth inhibition.

Clonogenic and migration-invasion assays’ methods can be found in the **Supplementary Material**.

### 2.3 Western blotting

Blots were incubated with i) primary antibodies against β-actin (MAB1501R, Millipore, 1:5000) paired with secondary horseradish peroxidase (HRP) antibody (#405306, BioLegend, 1:10000), ii) with primary antibodies anti-PARP (#9542S, 1:1000), and iii) anti-XIAP (#2045S, 1:1000) antibodies (Cell Signaling Technologies) paired with secondary HRP antibody (#31458, Invitrogen, 1:10000). For a detailed description, see **Supplementary Material**.

### 2.4 Albumin-based CuET nanoparticle synthesis

For *in-vivo* experiments, mouse albumin-based CuET nanoparticles (1 mg/mL) were synthesized as previously described (21). CuET powder is hardly soluble in any aqueous solution. Thus, for *in-vivo* experiments, mouse-albumin-based CuET nanoparticles (1mg/mL) were synthesized. Briefly, diethyldithiocarbamate (Sigma) (solved in water) is mixed with copper(ii) chloride (Sigma) (also in water) in a ratio 2:1. The reaction between these two compounds is carried in a 5% (w/v) mouse serum albumin (Innovative Research) solution. The resulting solution is stable at 4°C for at least 1 week. The dose administered to mice corresponded to the final concentration of 1 mg/kg of diethyldithiocarbamate-copper complex; the total injection volume was 0.5mL in isotonic salt.

### 2.5 Mice

8-week-old C57BL/6 and BALB/c mice received subcutaneous injections on the back, between the shoulder blades of 3.2 x 10^5^ MC-38 cells or 3 × 10^5^ CT-26 cells in 100μL PBS, respectively. Tumors became visible in the MC-38 and CT-26 grafts, ten- and seven-days following implantation on the backs of the mice, between the shoulder blades. Mice were randomized based on body weight into the following treatment groups: non-treated, vehicle (mouse albumin), and CuET (1 mg/kg). Treatment was administered through intraperitoneal injection (i.p.) as per the treatment schedules shown in respective figures. Tumor growth was monitored every second day through width (w) and length (L) measurements using an electronic calliper. Tumor volume (V) was calculated using the following formula: (V= W^2^ × L). Body weights were monitored every second day. Endpoint tumor volume was defined as 2 cm^3^ according to the FACC guidelines. Survival of the tumor-bearing mice was evaluated using Kaplan-Meier analysis. For the metastatic model, 8-week old C57BL/6 mice of mixed sexes were anaesthetized with isoflurane, 20 mg/kg carprofen and 0.1 mg/kg regular buprenorphine (s.c.) 30 min prior to surgery. Mice were placed in the right lateral recumbent position. The spleen was exposed through a small flank incision immediately below the rib cage (0.5 cm). 5x10^5^ MC-38 tumor cells were inoculated with a 28Gx1/2 U-100 0.5cc insulin syringe into the spleen and after 1 minute, the splenic arteries and venous supply were cauterized, and the spleen was removed. The mice were sutured using 5-0 Vicryl sutures. Hepatic cell and splenocyte cytotoxicity were evaluated on day 6 post-implantation of tumor cells in a subset of mice, whereas metastatic burden was assessed on day 14 in another subset. For metastatic burden analysis, livers were measured, and the total area was calculated as follows: (A= L*W). Metastatic lesions were counted, and the area occupied by the lesions was calculated according to the radius using: (A=πr^2^). All experimental procedures were performed in accordance with the Facility Animal Care Committee of McGill University Health Center, Montreal, QC, Canada.

### 2.6 Immunohistochemistry

24h after the last injection, the internal organs and tumors from the CuET-treated and control mice were removed, fixed with 10% buffered formalin for 48h, embedded in paraffin, and sectioned at 4µm. Hematoxylin and eosin (H&E) staining was performed. Immunostaining was performed using an automated immunostainer (Discovery XT; Ventana Medical Systems). Tumor tissue sections were stained with anti-CD3 (#05493315001, Roche), anti-CD19 (AB245235, Abcam), anti-F4/80 (#70076, Cell Signaling), anti-neutrophil elastase (PA5-79198, ThermoFisher), anti-MICA (100507436-MSM2-P0, ThermoFisher), and anti-ULBP1 (17715-1-AP, ThermoFisher) to stain T cells, B cells, macrophages, neutrophils, and NK cell ligands on cancer cells MICA and ULBP1. Scanning was performed using a Leica Aperio AT Turbo digital pathology scanner at 40X magnification and 25 microns/pixel. The infiltrating cells were quantified in QuPath-0.3.2, using ten fields per slide, data expressed as % positive cells per field, n=3 per group.

### 2.7 Fluorescence-activated cell sorting

For sorting, mouse hepatocytes from vehicle-treated mice and mice treated with 1 mg/kg CuET, were isolated in a 5.75 mL HBSS, 3.75 mL Percoll, 0.50 mL Heparin 1% in H_2_O gradient following the previously published protocol and stained fresh (43). Cells were stained with Fixable Viability Dye eFluor 506 (Invitrogen, 65086614, 1:1000). Extracellular staining with anti-CD3 APC-eF780 (Invitrogen, 47-0032-82, 1:200), anti-CD8b APC-Cy7 (YTS156.7.7, Biolegend,126620, 1:200), and anti-NK1.1 FITC (PK136, Invitrogen eBiosciences, 11-5941-85, 1:150) was performed. For NK and NKT cell enrichment, NK cells were defined as NK1.1+ and CD3 −, whereas NKT cells were defined as NK1.1+ and CD3+. Sorting was performed using a BD FACSAria III instrument.

### 2.8 Human PBMC isolation, NK and T cell cytotoxic assays

Human PBMC isolation and NK and T cell enrichment are described in detail in **Supplementary Material**. PBMC were incubated with autologous adherent cells and treated with or without 1nM CuET, to generate cytotoxic T cells, as previously described (44). Target tumor cells were maintained at 5 × 10^4^ cells. Four effector-to-target (E: T) ratios (20:1, 10:1, 5:1, and 2:1) were used for each assay. The Invitrogen™ CyQUANT™ LDH Cytotoxicity Assay Kit (ThermoFisher, C20301) was used to measure T and NK cytotoxic responses according to the manufacturer’s instructions. Lytic units were calculated using all four E:T ratios, as previously described (45).

### 2.9 Flow cytometry

Mouse organ harvesting and immunostaining was done as previously described (46). NK and T cells from human PBMC were first purified and enriched by negative selection as described in the **Supplementary Material**. After lymphocyte isolation, single-cell suspensions were stained with the following fluorescence-conjugated antibodies: NK and T cells untreated and treated with 1nM CuET were labeled with anti-human NKG2D (clone 1D11, ThermoFisher). Tumor cells were labelled with the NKG2D ligands: MICA (clone 159227, R&D Systems), ULBP1 (clone 170818, R&D Systems), and ULBP2/5/6 (clone 165903, R&D Systems). Antibody blocking experiments were performed using the same antibodies and the Thermo Scientific™ Pierce™ F(ab’)2 Preparation Kit (cat.44988). For perforin assessment from mouse splenocytes, the cells were fixed and permeabilized using the BDCytofix/Cytoperm™ Fixation/Permeabilization Kit (Cat.No.554714). Intracellular perforin staining with anti-perforin PE (S16009A, BioLegend, 154305, 1:100). Cells were acquired using a BD LSRFortessa flow cytometer (BD Biosciences) and analyzed on a FlowJo v.10 analysis platform (FlowJo, LLC).

### 2.10 RNA extraction and qPCR analyses

RNA was extracted using the Aurum Total RNA kit (Bio-Rad), and cDNA was produced using iScript RT Supermix RT-qPCR (Bio-Rad) from 2 μg of total RNA in a 40 μl RT reaction. Quantitative real-time PCR was performed using the SSoFast EvaGreen Supermix (Bio-Rad) at an annealing temperature of 55°C. The final concentration of primers was 400 nM, and 30 ng (3 μl) of cDNA was used in a 10 μl qPCR reaction volume. Primer sequences for human ULBP1 (NM_025218), ULBP2 (NM_025217), MICA (NM_000247), MICB (NM_005931), and β-tubulin (housekeeping gene) mRNAs are listed in **Supplementary Table 1**. The fold-change was calculated using the formula 2^(−ΔΔCt)^.

### 2.11 The Cancer Genome Atlas analyses

The projects TCGA-READ (rectal adenocarcinoma), TCGA-COAD (colon adenocarcinoma), and TCGA-BRCA (breast carcinoma) were downloaded using the R package TCGAbiolinks v2.25.3. Outlier samples were detected using arrayQualityMetrics before and after gene count normalization using edgeR. Samples were considered outliers and removed if they were marked as outliers before and after normalization using the same metrics or if they were marked as outliers by multiple metrics after normalization (COAD n=8; READ n=1; BRCA n=13). arrayQualityMetrics uses three metrics to mark outliers: 1) the total distance between a sample and all other samples, 2) the Kolmogorov-Smirnov test between a sample’s distribution and the pooled distribution of all other samples, and 3) Hoeffding’s statistic to determine sample independence. Highly variable genes were selected based on their median absolute deviations (MAD). For COAD and READ, genes with MAD ≥ the 55th most variable gene (MAD=0.033) were retained. For BRCA, genes whose MAD was ≥ the 50th most variable gene (MAD=0.022) were retained. Overall, between 27,300 and 30,321 genes remained for analyses. Differential expression analyses of the 11 candidate genes were performed using edgeR: ULBP1, ULBP2, ULBP3, MICA, MICB, CD274, RAET1L, RAET1G, HCST, CD276, and KLRK1. Differentially expressed genes had a Bonferroni Correction adjusted *p* < 0.05.

### 2.12 Statistical analyses

Statistical analyses were performed using the GraphPad Prism 9 software (GraphPad Software, San Diego, CA, USA). One-way ANOVA or two-way ANOVA tests with Dunnett’s or Holm-Sidak corrections were employed for multiple comparisons involving either a single variable or multiple variables, respectively. When comparing only two conditions for a single variable, a *t*-test was used with either Welch’s correction or Sidak’s correction. Data are presented as the mean ± SD unless otherwise indicated (*p<0.05, **p<0.005, ***p<0.0005, and ****p<0.0001).

## 3 Results

### 3.1 CuET is cytotoxic in murine and human hypermutated and non-hypermutated CRC cell lines and induces apoptosis *in vitro*

**Figure 1:**
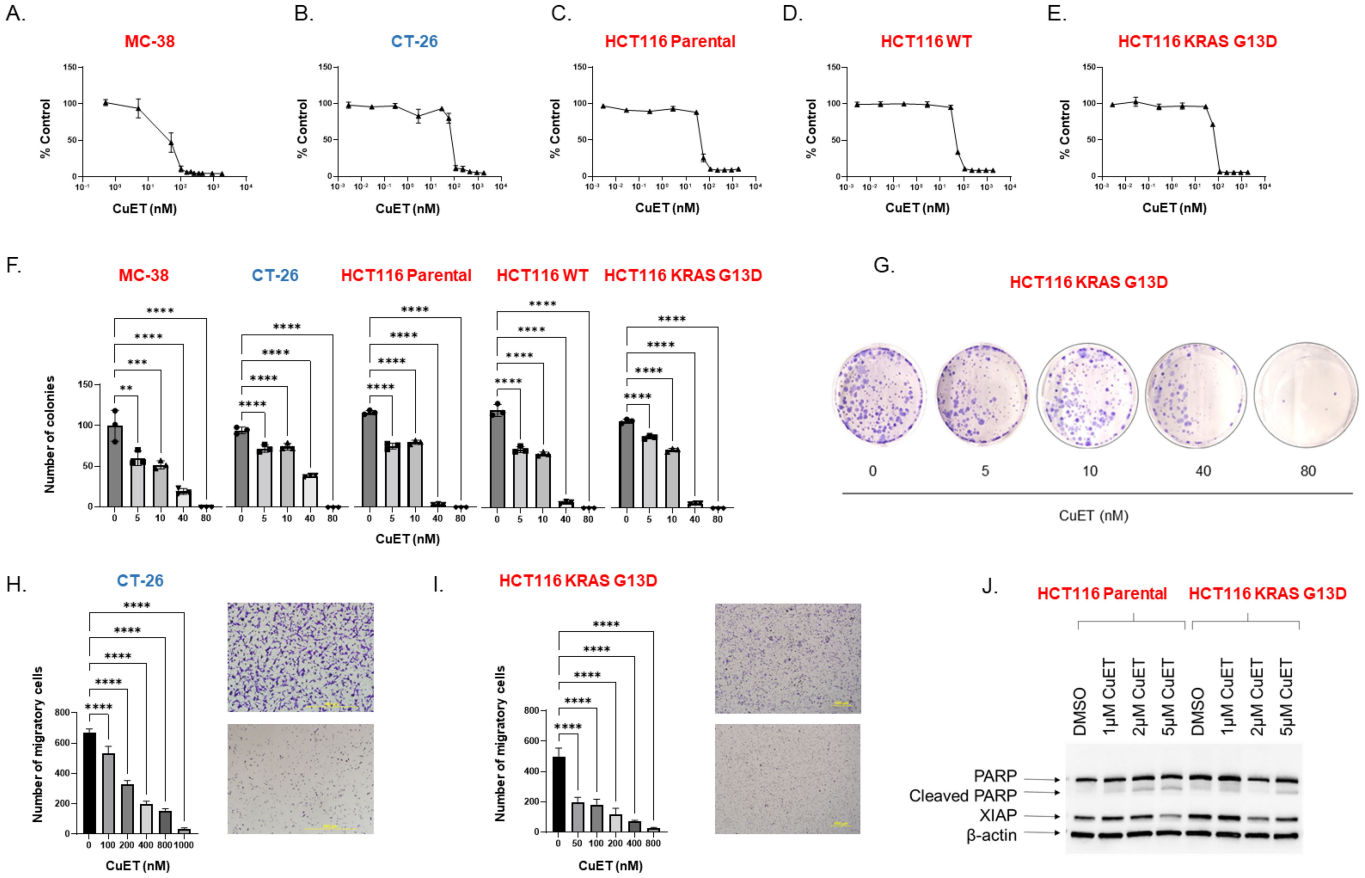
CuET is cytotoxic, induces apoptosis, and inhibits migration-invasion of CRC *in vitro.* **(A-E)** CRC cell lines MC-38, CT-26, HCT116 parental (KRAS^G13D^/KRAS^WT^), HCT116 WT (KRAS ^WT^/KRAS ^KO^), and HCT116 KRAS^G13D^ (KRAS^G13D^/KRAS ^KO^) were treated with serial dilutions of CuET (0nM to 1.8μM) for 72 h, and cytotoxicity was evaluated by Sulforhodamine B assay. IC50 values of CuET were 45.4nM and 68.2nM for MC-38 and CT-26, respectively, and 54.8nM, 54.0nM, and 48.3nM for HCT116 parental, WT, and KRAS^G13D^ cell lines. Cell lines in red are MMRd, MSI and in blue are MMRp and MSS. **(F)** Colony formation assays were performed after treating CRC cell lines with CuET (5-80nM) for seven consecutive days. Data represents three independent experiments. Ordinary One-Way ANOVA with Dunnett’s multiple comparison test where **p<0.005, ***p<0.0005 and ****p<0.0001. **(G)** Representative image of HCT116 KRAS G13D colony formation assay. **(H, I)** Migration and invasion inhibition by CuET was assessed in CRC cell lines CT-26 and HCT116 KRAS^G13D^ plated in Boyden-chamber assay and treated with CuET (0-1µM) for 24h (Scale bar 500um). Data represents three independent experiments. Ordinary One-Way ANOVA with Dunnett correction where **p<0.005, ***p<0.0005 and ****p<0.0001. **(J)** Western blot analysis illustrates the expression of cleaved PARP and XIAP in cell lysates of HCT116 parental and KRASG13D treated with CuET (0-5μM) for 24h. β-actin was used as the internal control.

Direct cytotoxicity of CuET was assessed *in vitro* using murine CRC cell lines MC-38 and CT-26 and human HCT116. MC-38 harbors a KRAS mutant and MSI/MMR-d and is a cell line model of an immune hot tumor. CT-26 do not express a mutation at the KRAS locus (wild-type) and are MSS/MMR-p, modeling an immune cold tumor phenotype. CuET inhibited cell viability in a dose-dependent with mean half-inhibitory concentrations (IC50) of 45.4nM and 68.2nM for MC-38 and CT-26, respectively (**Figures 1A, B**). In HCT116 cell lines, the parental cell line heterozygote for mutant KRAS at position G13D, displayed an IC50 of 54.8nM, the wild-type KRAS cell line had an IC50 of 54.0nM, and the homozygous mutated KRAS^G13D^ cell line had an IC50 of 48.3nM (**Figures 1C–E**). Additionally, CuET inhibited the survival and proliferation of all CRC cell lines in a dose-dependent manner, with significant inhibition occurring at 5nM, as demonstrated by the clonogenic assay (**Figures 1F, G**). Finally, the migration-invasion potential of the cells was assessed in a Boyden chamber, where CuET inhibited cell migration and invasion through a collagen matrix at doses ranging from 50 to 800nM in HCT116 KRAS^G13D^ cells and 100nM to 1µM in CT-26 cells (**Figures 1H, I**). Cell death by apoptosis was confirmed by PARP cleavage and XIAP degradation in HCT116 parental and HCT116 KRAS^G13D^ cells (**Figure 1J**).

### 3.2 Systemic administration of albumin-CuET nanoparticles is safe in mice

*In vivo* safety assessment was performed by administering the albumin-CuET nanoparticle formulation (1 mg/kg) to 8-week-old BALB/c mice every three days over 17 days. Monitoring revealed no adverse events or deaths during treatment. CuET injections did not affect body weight (**Supplementary Figure 1A**), indicating that it was well tolerated *in vivo*. Blood biochemical parameters in BALB/c mice, including liver and renal functions, remained within the reference ranges and matched vehicle-treated and untreated animals, as evidenced by normal plasma AST, ALT, and creatinine levels (**Supplementary Table 2**).

Histological examination revealed no internal organ toxicity, with no morphological changes in liver, kidneys, heart, or spleen compared to controls (**Supplementary Figure 2**). Absence of vascular congestion, fatty changes, and inflammatory changes ruled out the possibility of hepatotoxicity. Renal toxicity was excluded because of the absence of glomerular atrophy and tubular necrosis. Myocardial hypertrophy, edema, or interstitial tissue fibrosis were not observed. Spleen assessment showed no hyperplasia or hypoplasia of the white or red pulp. Erythrocyte, platelet, and white blood cell counts remained consistent across the treatment and control groups (**Supplementary Figure 3**). These findings collectively establish the excellent safety profile of CuET nanoparticles.

### 3.3 Systemic treatment with albumin-CuET nanoparticles significantly inhibited tumor growth and induced apoptosis in a CT-26 syngeneic model of colorectal cancer in BALB/c mice

**Figure 2:**
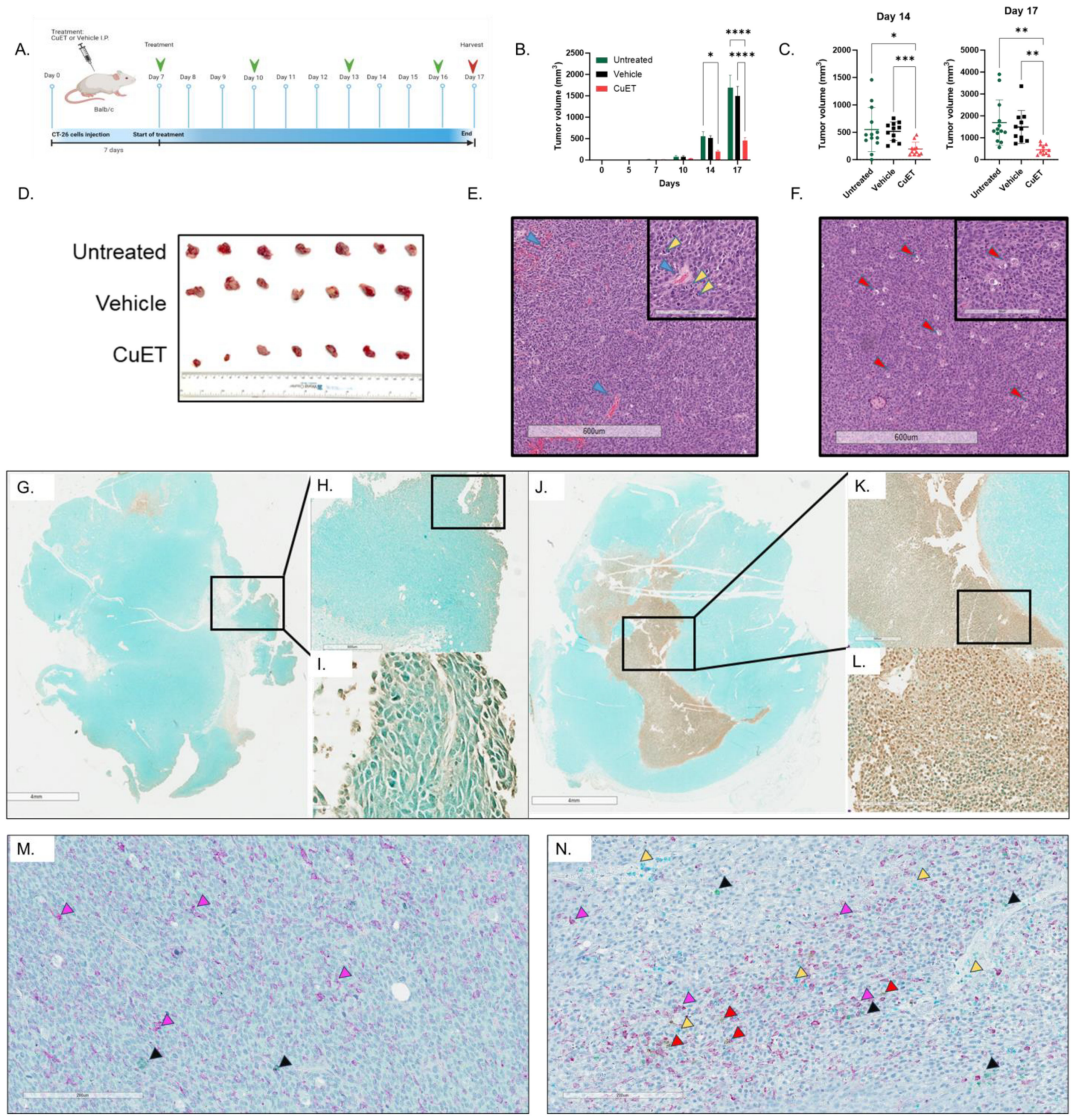
CuET inhibits CT-26 cold CRC tumor model in mice by inducing apoptosis and immune cell infiltration. **(A)** Experimental design representing sub-cutaneous injection of CT-26 cells on day 0 in BALB/c mice. Treatment with 1mg/kg CuET (n=11), vehicle (n=11), and untreated (n=13) was administered as indicated by green arrows. **(B)** Tumor growth inhibition is observed throughout the treatment, with statistically significant differences observed on day 14 and 17. Data expressed as mean ± SEM. Tukey’s Two-Way ANOVA where *p<0.05, **p<0.005, ***p<0.0005 and ****p<0.0001. **(C)** Individual data points representing tumor measurements at days 14 and 17. Data expressed as mean ± SD. Welch ANOVA with Dunnett’s T3 correction where *p<0.05, **p<0.005, ***p<0.0005 and ****p<0.0001. **(D)** Visual representation of excised tumors on day 17. **(E, F)** Hematoxylin and eosin stain of representative sections from tumors of an untreated and a CuET-treated mouse, respectively, at 4X (scale= 600um) and 20X (inlet, scale= 200um) magnifications. **(G-I)** TUNEL assay showing apoptotic cells in brown (DAB) in the tumor section of a vehicle-treated mouse at 1X (scale= 4mm), 4X, and 40X magnification. **(J-L)** TUNEL assay showing apoptotic cells in brown (DAB) in the tumor section of a CuET-treated mouse at 1X (scale= 4mm), 4X, and 40X magnification. **(M)** Immunohistochemistry, 20X (scale= 200um) magnifications of tumor sections from a vehicle-treated mouse. **(N)** Immunohistochemistry, 20X (scale= 200um) magnifications of tumor sections from a CuET-treated mouse. Antibodies against macrophages (F4/80 stained purple) indicated by purple arrows, against neutrophils (neutrophil elastase-stained teal) indicated by yellow arrows, against T cells (CD3 stained green) indicated by black arrows, and against B cells (CD19 stained brown/DAB) indicated by red arrows.

The therapeutic efficacy of albumin-CuET was evaluated in a syngeneic ectopic CT-26 CRC cold tumor model in BALB/c mice (**Figure 2A**). After three treatments, CuET significantly inhibited tumor growth by day 14, maintaining this difference until the end of the experiment on day 17 (**Figures 2B, C**). H&E staining revealed distinct morphological differences. Untreated tumors (**Figure 2E**) exhibited partial necrosis and poorly differentiated adenocarcinomas with pronounced vascularity and hemorrhage (blue arrows). Observational histological analysis identified numerous mitotic figures with elongated, aligned chromosomes (yellow arrows). In contrast, observational histological analysis of CuET-treated tumors showed an increase in vacuolated (red arrows), potentially senescent and dying, tumor cells (**Figure 2F**). TUNEL staining confirmed CuET-induced apoptosis, revealing increased apoptotic cells at the core of tumors in CuET-treated mice compared with vehicle-treated mice (**Figures 2G–L**).

### 3.4 Systemic treatment with albumin-CuET nanoparticles significantly increases T cell and macrophage infiltration inside tumor cores in a CT-26 syngeneic cold tumor model of colorectal cancer in BALB/c mice

Immunohistochemical analysis of excised tumors was performed to evaluate the cell populations within the tumor cores (**Figures 2M, N**). CuET treatment did not significantly enhance neutrophil or B-cell infiltration compared to vehicle treatment. However, CuET-treated mice exhibited significantly increased recruitment of macrophages and T cells to the tumor cores compared with the vehicle (**Supplementary Figure 4**).

### 3.5 Systemic treatment with mouse albumin-CuET nanoparticles inhibited ectopic tumor growth, liver metastasis, and increased survival in an MC-38 syngeneic hot tumor model of colorectal cancer

**Figure 3:**
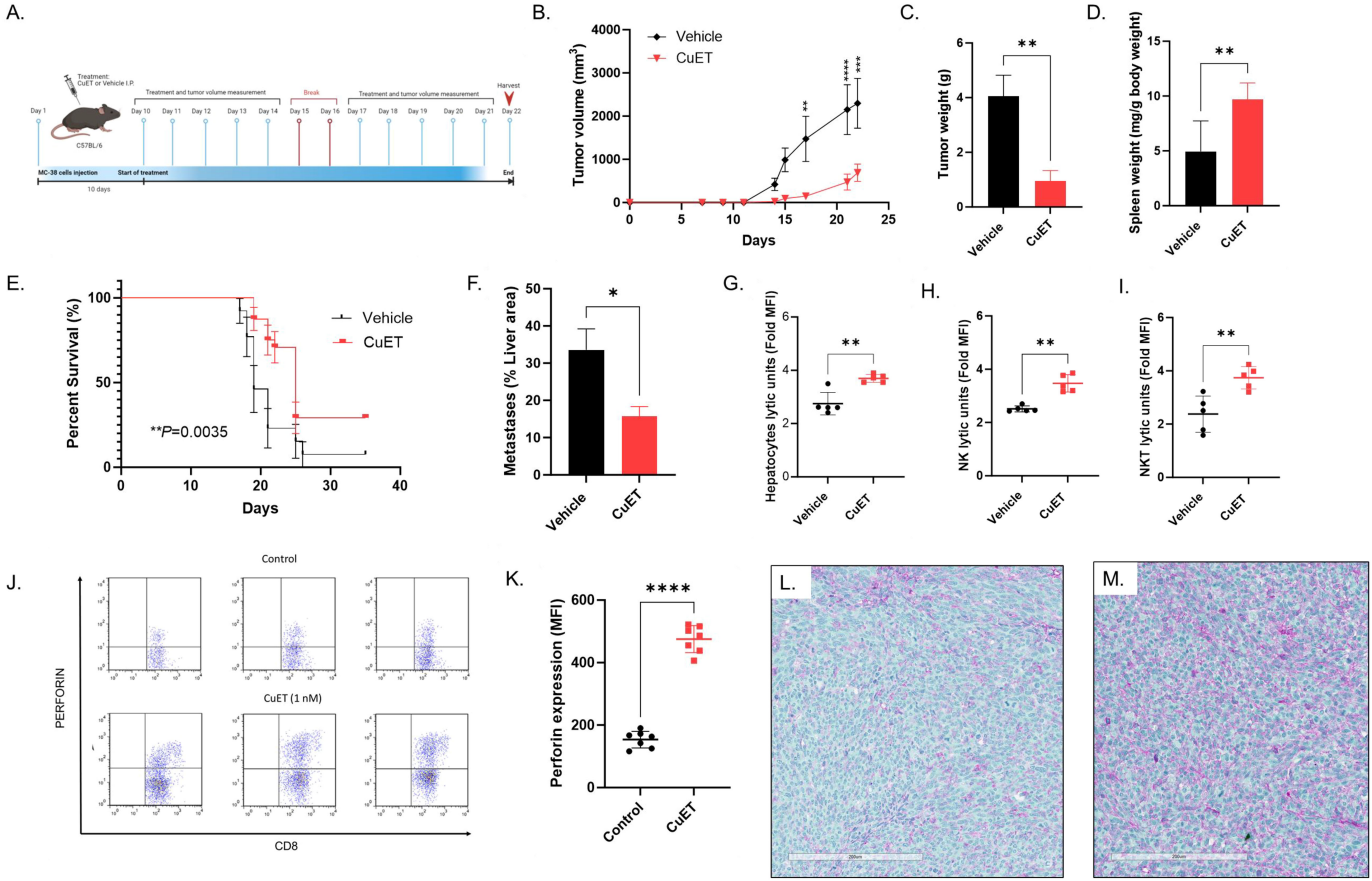
CuET inhibits metastatic CRC growth, prolongs survival, and activates NK and NKT cells in MC-38 mouse model. **(A)** Experimental design representing sub-cutaneous injection of MC-38 cells on day 1 in C57BL/6 mice. Treatment with 1mg/kg CuET and the vehicle was administered i.p. as indicated. **(B)** Tumor growth inhibition in the ectopic model is observed throughout the treatment with CuET (n= 6 per group). Statistically significant differences were observed on day 17. Data expressed at mean ± SEM. Sidak’s Two-Way ANOVA. **(C)** Tumor weight at the endpoint, day 22. Data expressed at mean ± SEM. Welch’s t-test. **(D)** Spleen weight at the endpoint, day 22. Data normalized as mg of spleen per g of body weight expressed at mean ± SD. Welch’s t-test. **(E)** Kaplan-Meier analysis of mouse survival in CuET-treated (n=24) versus vehicle-treated (n=13) mice. Log-rank (Mantel-Cox) test. **(F)** Liver area occupied by MC-38 metastatic lesions at day 14 (n=5 mice per group), data expressed as mean ± SEM. Welch’s t-test. *p<0.05, **p<0.005 and ****p<0.0001. **(G-I)** C57BL/6 mice with liver metastatic MC-38, treated with CuET or vehicle, every day for five days, were harvested at day 6 post-implantation. Respectively hepatocytes, and FACS enriched-NK cells or -NKT cells lytic units in co-culture with MC-38 cells for 24h, data expressed as mean ± SD of fold increase in median fluorescence intensity (MFI) of green, fluorescent objects indicating cell death. Data normalized to time point 0. **(J)** Three representative scatterplots of perforin expression in CD8+ mouse splenocytes, n=3 per group, respective MFIs from left to right: Control 95.9 vs CuET 400, Control 133 vs CuET 325, Control 195 vs CuET 425. **(K)** Perforin expression is increased by CuET stimulation of C57BL/6 mouse splenocytes *ex-vivo* for 18h, n= 7 per group, data expressed as mean ± SD. **(L)** Immunohistochemistry, 20X (scale= 200um) magnifications of MC38 tumor sections from a vehicle-treated mouse. **(M)** Immunohistochemistry, 20X (scale= 200um) magnifications of MC38 tumor sections from a CuET-treated mouse. Antibodies against NK cell cancer ligands MICA (purple) and ULBPs (green).

The therapeutic effect of CuET was assessed in MC-38 syngeneic ectopic and metastatic CRC hot tumor models in C57BL/6 mice (**Figure 3A**). Statistically significant inhibition of ectopic tumor growth was observed in the CuET group after five systemic treatments, with sustained differences in tumor volume until the end of the experiment on day 22 (**Figure 3B**) and reduced tumor weight at endpoint (**Figure 3C**). Interestingly, CuET-treated mice exhibited enlarged spleens (**Figure 3D**). Kaplan-Meier analysis indicated significantly prolonged survival in CuET-treated mice compared with vehicle-treated mice, with a median survival of 25 and 19 days, respectively (**Figure 3E**). In the metastatic model, CuET reduced liver metastatic lesions, occupying 15.73% of the liver area compared to 33.56% in controls after 14 days (**Figure 3F**). Overall, CuET inhibited tumor growth in syngeneic ectopic and metastatic models, and prolonged survival in mice.

### 3.6 NK and NKT cells of mice treated with albumin-CuET nanoparticles display enhanced cytotoxicity against the mouse MC-38 colorectal cancer cell line *ex vivo*

Following the efficacy of CuET in inhibiting MC-38 metastatic tumor growth in the mouse liver, we explored the *ex-vivo* immune cytotoxicity of hepatocytes. CuET treatment of mice for six days increased *ex-vivo* cytotoxicity of hepatocytes against MC-38 cells over 24h (**Figure 3G**). This effect was accentuated in enriched cultures of NK cells (**Figure 3H**) and NKT cells (**Figure 3I**), demonstrating a lytic capacity approximately 2-fold higher than that of vehicle-treated mice. Significant differences were observed between 12h and 14h after co-culture (data not shown) and were sustained for 24h. Intracellular staining of mouse splenocytes stimulated *ex-vivo* with 1nM CuET revealed increased production of the effector cytokine perforin by CD8+ NK and T lymphocytes, compared to control (**Figures 3J, K**). Gating strategy can be found in **Supplementary Figure 5**. Immunohistochemical staining of MC-38 tumors revealed positive staining for MICA (purple) and ULBP1 (green) ligands in both vehicle and CuET-treated groups (**Figures 3L, M**).

### 3.7 CuET increases human NK and T cell cytotoxicity against HCT116 and HT-29 colorectal cancer cell lines through surface expression of the activating receptor NKG2D on lymphocytes and cancer ligands

**Figure 4:**
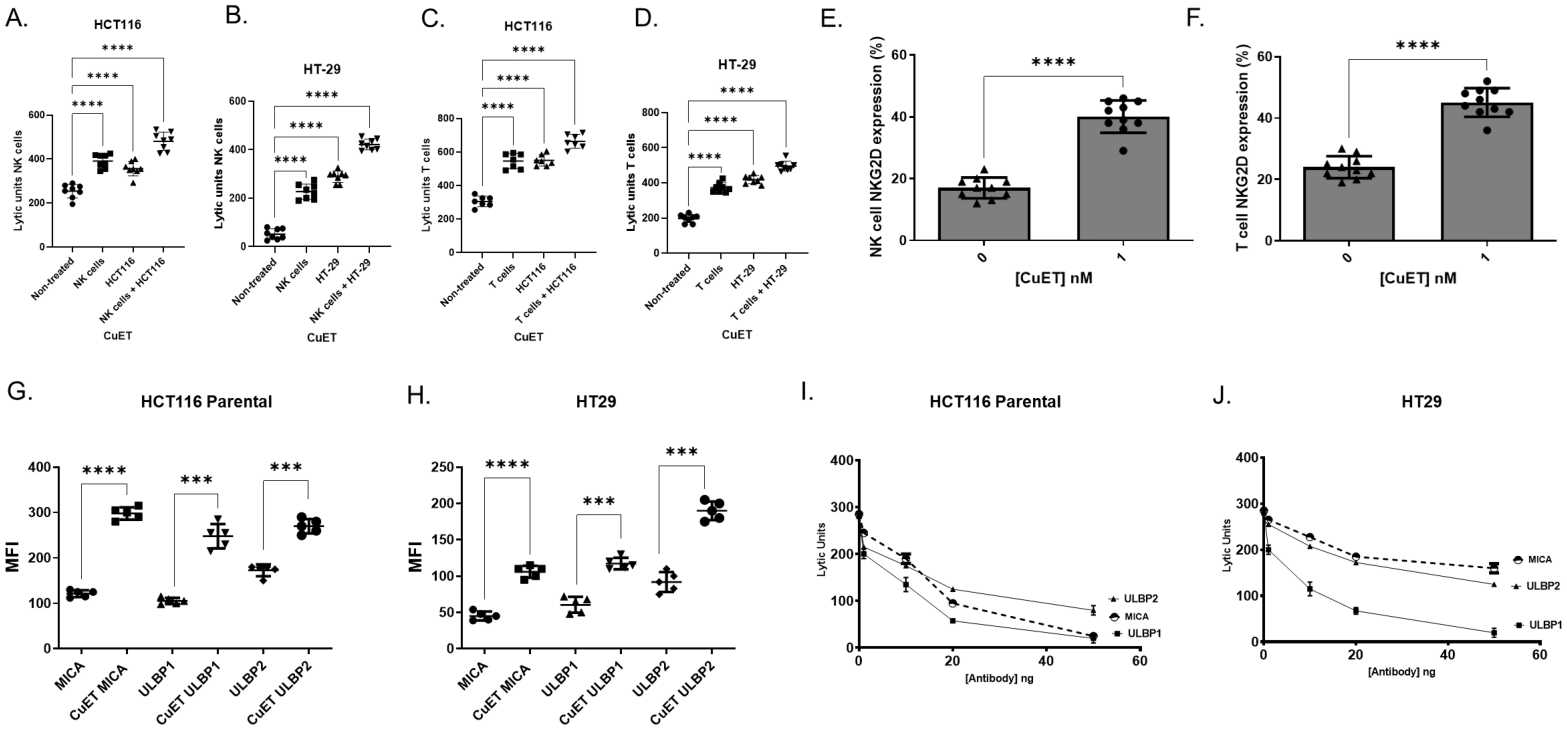
CuET increases human NK and T cell cytotoxicity and surface expression of NKG2D and ligands. NK and T lymphocytes were purified by negative selection from human PBMC. A-D) NK lytic units **(A, B)** and T cell lytic units **(C, D)** against HCT116 parental and HT-29 cells. Non-treated lymphocytes are compared to 1nM CuET for 18h pre-treated NK or T cells (effector), HCT116 parental or HT-29 cells (target), and simultaneously pre-treated effector and target cells. Data expressed as mean ± SD, n= 8 per group, One-Way ANOVA, where *p<0.05, **p<0.005, ***p<0.0005 and ****p<0.0001. **(E, F)** Surface expression (%) of NKG2D receptor in CuET treated and untreated NK and T lymphocytes. Data expressed as mean ± SD, n= 10 per group, Welch’s t-test where ***p<0.0005, and ****p<0.0001. **(G, H)** Surface expression of MICA, ULBP1, and ULBP2 in HCT116 parental and HT-29 human CRC cells treated with 1nM CuET for 18h versus DMSO control. Data expressed as mean fluorescence intensity ± SD of n=5 per group. **(I, J)** NK cell functional assay against HCT116 parental and HT-29 CRC cells stimulated with 1nM CuET for 18h, where NKG2D ligands MICA, ULBP1, and ULBP2 were blocked with 0, 1, 10, 20, and 50 ng of antibody. Data expressed as mean ± SD of n=3 per group.

The ability of CuET to stimulate NK and T lymphocytes (effector) purified (**Supplementary Figures 6A, D**) from human PBMCs co-cultured with HCT116 and HT-29 cells (target) was assessed. Baseline cytotoxicity was seen in untreated NK and T cells (**Figures 4A–D**). Treatment with 1nM CuET significantly boosted NK and T cell cytotoxicity (**Figures 4A, B**). The same effect was observed when T cells were treated with CuET (**Figures 4C, D**). Additionally, target cell treatment with CuET maintained effector cell cytotoxicity at the same level as when only the effector cells were treated. Interestingly, an additive effect was observed when both effector and target cells were stimulated with CuET before co-culture, suggesting a two-fold effect: lymphocyte activation, enhanced immunogenicity, and targeting of cancer cells. These results prompted the characterization of the surface receptors on lymphocytes and their respective cancer cell ligands.

Flow cytometry analyses revealed increased NKG2D receptor expression on NK and T lymphocytes treated with 1nM CuET (**Figures 4E, F**) (**Supplementary Figures 6B, C, E, F**), as well as a significant increase in NKG2DLs MICA, ULBP1, and ULBP2 on human CRC cell lines HCT116 parental and HT-29, treated with 1nM CuET (**Figures 4G, H**). At the mRNA level, the modulation of NKG2D ligands in HCT116 parental cells under treatment with 1µM CuET induced a 3-fold and almost 4-fold increase in expression of ULBP1 and MICB, respectively, at 6h, and a 2.5-fold increase in ULBP2 expression at 24h (**Supplementary Figures 7A–D**). Time-dependent gene modulation was also observed in the HCT116 G13D cell line (**Supplementary Figures 7E–H**).

The importance of ligand expression in tumor cells previously stimulated with CuET prior to NK challenge was confirmed through antibody blocking of these ligands on cells, effectively decreasing the functional activity of NK cells in a dose-dependent manner, as evidenced by the inversely proportional decrease in cell cytotoxicity as antibody concentrations increased (**Figures 4I, J**). In HCT116 cells, the inhibition of NK cell cytotoxicity was higher when blocking MICA and ULBP1 (approximately 90% at 50ng) and less effective when blocking ULBP2 (75% at 50ng). In contrast, for HT-29 cells, inhibition of ULBP1 resulted in a 98% decrease in cytotoxicity at 50ng of antibody, whereas only a 50% and 43% decrease was observed when ULBP2 and MICA were blocked, respectively. The efficacy of antibody blocking with Fab fractions is shown in **Supplementary Figure 8**. Correlation analysis between antibody blocking of NKG2D ligands and lytic units of NK cells against cancer cells was performed. Respectively, R^2^ of 0.56 for MICA, 0.93 for ULBP1, and 0.88 for ULBP2 were obtained for HCT116 cells (**Supplementary Figure 8G**). Similarly, R^2^ of 0.30 for MICA, 0.73 for ULBP1, and 0.60 for ULBP2 were obtained for HT29 (**Supplementary Figure 8H**).

### 3.8 NKGD2L mRNA expression is elevated in tumor samples across three cohorts of cancer patients

**Figure 5:**
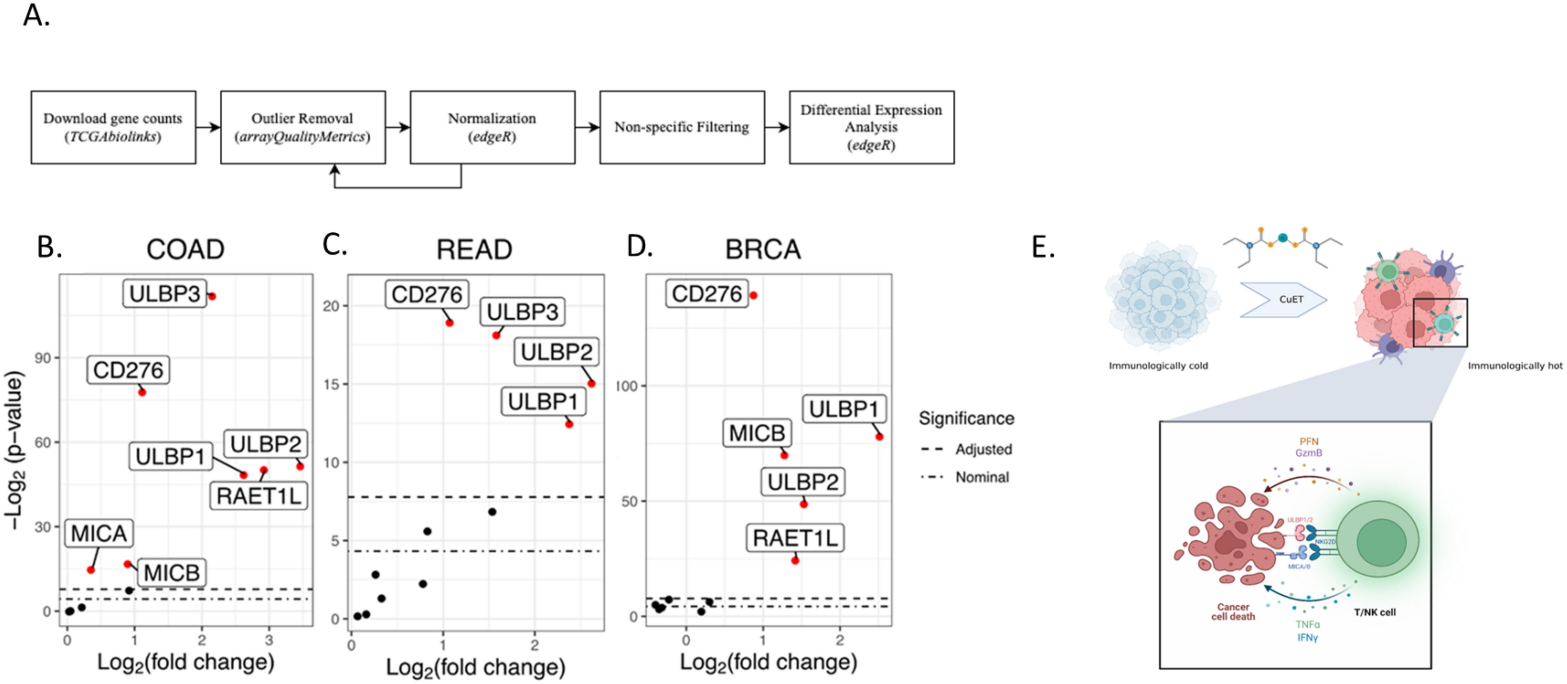
Comparative analysis of NKG2DL expression in tumor samples versus normal tissue across three TCGA projects. **(A)** Flowchart of differential expression analysis methods. **(B-D)** Summary boxplots identifying differentially expressed genes in solid tissue, normal samples, versus primary tumour samples across three cancer data sets. Differentially expressed genes are shown in red (COAD n=7; READ n=4; BRCA n=5). The upper bound dashed line indicates the Bonferroni Correction significance threshold. The lower bound dashed line indicates the nominal significance threshold (p = 0.05). **(E)** Schematic of immunoadjuvant mechanism of action of CuET.

Differential gene expression analysis across tumor samples and adjacent normal tissues from the COAD (colon adenocarcinoma), READ (rectum adenocarcinoma), and BRCA (breast carcinoma) projects of The Cancer Genome Atlas Program was performed after outlier removal and normalization (**Figure 5A**; **Supplementary Figure 9**). The analysis revealed an upregulation of NKG2D ligands in the tumor tissue (**Figures 5B–D**). Among 11 candidate genes, seven genes in the COAD dataset, four genes in the READ dataset, and five genes in the BRCA dataset were upregulated in the tumor tissues compared to normal tissue. The COAD tumor samples showed an increase between 4.5 and 11.3-fold for ULBP1/2/3/6 compared to normal tissues, and MICA and MICB had expression increases of 1.29 and 1.89-fold (**Supplementary Figure 10A**). READ tumors showed 3.03 to 6.06-fold increases in ULBP1/2/3 expression (**Supplementary Figure 10B**). BRCA tumors had 2.82 to 5.65-fold increases in ULBP1/2 expression, along with upregulation of RAET1L and MICB by 2.63 and 2.46-fold, respectively (**Supplementary Figure 10C**). Notably, MICA was significantly upregulated in tumors of the COAD but not BRCA dataset, while MICB was significantly upregulated in tumors of both COAD and BRCA. All three datasets saw a statistically significant upregulation of ULBP1/2 and CD276 in tumor samples versus adjacent normal tissue.

## 4 Discussion

Despite advancements in cancer screening, colorectal cancer (CRC) remains a significant cause of mortality, claiming nearly one million lives annually. While surgery is the mainstay treatment, adjuvant therapies such as chemotherapy and radiation have improved disease-free survival rates reaching 78.2% with varying regimens (47). Immunotherapy, including immune checkpoint inhibitors (ICI), monoclonal antibodies, adoptive cell therapy (CAR-T and CAR-NK therapies), and oncolytic virus treatment, has shown promise in recent years (48). However, the efficacy and toxicity of these therapies vary widely among patients due to CRC’s mutational heterogeneity and diverse immune profiles (49).

The correlation between elevated tumor-infiltrating lymphocytes (TILs) and immunotherapy response has been established, with low TIL levels often indicating non-responsiveness (50–52). Conversely, high levels of cytotoxic (CD8+) T cells and NK cells correlate with favorable prognosis and increased survival (53). This categorization of tumors as ‘cold’ or ‘hot’, shapes strategies to transform cold tumors into hot ones for broader immunotherapy efficacy (54). The established framework for antitumor immunity hinges on CD8+ T cells recognizing specific antigens presented by target cell major histocompatibility complex class I (MHC-I) molecules, resulting in tumor cell elimination. While the traditionally described pathway of tumor immune evasion involves the downregulation of tumor MHC-I, recent studies have shown that CD8+ T cells retain the ability to eliminate tumor cells, even in the absence of MHC-I expression. Indeed, Lerner et al. demonstrated that CD8+ T cells maintain cytotoxic function through the T cell NKG2D receptor and tumor NKG2D ligands, which are particularly abundant on MHC-deficient variants (55). Importantly, CD8+ T cell priming without CD4+ T cell co-stimulation generates ‘helpless’ CD8+ T cells with diminished effector functions and minimal memory formation. However, their studies have shown that NKG2D signaling can provide immunological assistance to CD8+ T cells, rescuing their effector and memory functions (55). These findings suggest that NKG2D signaling in CD8+ T cells goes beyond established canonical functions, such as aiding target recognition and promoting killing, by supporting cell survival (56). Clinical data indicating improved survival in CRC patients with increased T cell and NK cell tumor infiltration emphasizes the potential of therapies that enhance both the quantity and function of TILs.

In this study, we investigated the immunomodulatory role of the metabolite copper-diethyldithiocarbamate (CuET) in two mouse models, including a syngeneic metastatic model of CRC. CuET exhibited strong *in vitro* cytotoxic effects with nanomolar IC50 values across multiple cancer cell lines, irrespective of mutations (**Figure 1**). CuET treatment significantly increased mouse survival and inhibited tumor growth in ectopic and metastatic models (**Figures 2**, **3**). Moreover, CuET maintained a favorable safety profile while promoting the recruitment of CD3+ lymphocytes to the TME (**Figure 2**).

Functional analyses of liver cell populations from mice harboring CRC liver metastases treated systemically with albumin-CuET nanoparticles revealed enhanced NK and NKT cell cytotoxicity against CRC cells, partially explaining the antimetastatic activity of the drug (**Figure 3**). *Ex vivo*, CuET induced the expression of the effector cytokine perforin in mouse CD8+ lymphocytes (**Figures 3J, K**), highlighting tumor control through NK and T cell cytotoxicity.

Functional enhancement of NK and T cells by CuET was confirmed in human PBMC, where *ex vivo* stimulation with 1nM CuET not only increased the lytic activity of these lymphocytes against CRC cells but also significantly enhanced the surface protein expression of the NKG2D receptor (**Figure 4**). NKG2D is a type II C-type lectin-like transmembrane protein, and an activating receptor expressed on the surfaces of NK cells, NKT cells, activated CD8+ T cells, and a subset of γδ T cells. It plays a role in the transduction of an activation signal upon recognition of receptor ligands of the unique long 16-binding protein (ULBP) family and MICA/B on the surface of cancer cells. The NK and NKT circulating cells of patients with CRC have been reported to express significantly lower levels of NKG2D activating receptors (57). Meanwhile, studies have demonstrated the prognostic value of NKG2D ligands. In breast cancer, MICA/B ligands and ULBP2 are associated with a longer relapse-free period (58). Interestingly, in the context of CRC, elevated expression of all ligands was frequent in tumor-node-metastasis stage I tumors but was seen less frequently in more advanced tumors, indicating an immunoediting mechanism favoring the survival of tumor cells harboring diminished or absent expression of NKG2D ligands (59).

Furthermore, NKG2D ligands (NKG2DLs), including ULBP family members, MICA, and MICB, are often upregulated in tumors compared to healthy tissues, rendering them an interesting focus for immune targeting (60). TCGA differential expression analysis of three cancer types cohort analyses, TCGA-COAD, TCGA-READ, and TCGA-BRCA, confirmed that NKG2DLs were consistently elevated in tumor tissues as compared to healthy adjacent tissues (**Figures 5B–D**). When examining the effect of CuET on NKG2DLs expressed in human tumor cell lines (HCT116 and HT-29), we observed effective upregulation of MICA/B and ULBP1/2 at the mRNA and protein levels (**Figure 4**), (**Supplementary Figure 7**). Valés-Gómez et al. have previously shown that proteasome inhibitors induce NKG2DL expression in tumor cells. Considering that CuET effectively inhibits the p97-NPL4 complex, resulting in strong proteasome inhibitory effects and inducing heat shock response through heat shock protein 70 (HSP70), as reported by Skrott et al., this effect was not surprising (21, 61). Our results confirmed the role of CuET in modulating the NKG2D receptor-ligand binding axis, demonstrated by selective antibody blocking of cancer cell ligands and correlated dose-dependent decrease in NK-specific lysis activity (**Figures 4I, J**), (**Supplementary Figure 8**). NKG2D binding to its ligands on tumor cells activates NK cells, enhances cytotoxicity, and stimulates IFNγ production, facilitating a robust anticancer immune response (62).

Taken together, these data support that CuET stimulates i) NKG2D receptor expression on NK and T cells and ii) NKG2D ligand expression, such as MICA/B and ULBP1/2, on tumor cells, thereby facilitating immune recognition and more effective elimination of tumor cells by cytotoxic effector cells (**Figure 5E**).

An interesting approach to harness NK cell-driven tumor killing involves adoptive cell transfer, with recent advancements in CAR-NK cells engineered to express receptors targeting specific tumor antigens for enhanced tumor killing and co-stimulation of T lymphocytes. Most clinical trials have focused on the treatment of hematological cancers, using allogeneic NK cells or NK-92 cell lines (63). These approaches have limitations such as short *in vivo* persistence post-infusion. In contrast, CAR NK cells have demonstrated efficient tumor cell killing *in vivo*. For instance, Xiao et al. demonstrated significant inhibition of CRC progression in a xenograft mouse model using chimeric NKG2D CAR-NK cells (64). In the same study, results from three patients with refractory metastatic CRC treated with a local infusion of NKG2D CAR-NK reduced the number of tumor cells in the ascites of the patients, and regression was observed in the metastatic liver lesions of one patient. Critical challenges in CAR-NK therapy involve enhancing NK cell cytotoxicity against tumors and increasing their abundance and persistence within the TME.

Noteworthily, CRC involves immune evasion pathways beyond PD-1/PD-L1 and CTLA4, with emerging targets like LAG3, TIM-3, and TIGIT under clinical investigations (65, 66). Single checkpoint blockade may induce compensatory upregulation of other checkpoint receptors, exemplified by the upregulation of LAG-3 and CTLA-4 with anti-PD-1 mAb treatment in mice, suggesting a need for combination therapies (67). In addition to the combination of immune checkpoint inhibitors, the integration of immunoactivating drugs such as CuET offers a compelling approach to target the immune system in a dual manner. Small immunomodulating molecules targeting the NKG2D-NKG2DL axis could enhance the shift in phenotype towards an immune-responsive profile, even in CRCs lacking MHC-I expression and/or adequate CD4+ T cell co-stimulation.

In summary, this study is the first to demonstrate the immunomodulatory properties of CuET in NK-and T cell-mediated tumor killing. Limitations include challenges in determining the activation effects of CuET on peripheral versus tumor-associated lymphocytes in cancer patient-samples. Nonetheless, the findings significantly enhance our understanding of the influences of CuET on anticancer immunity, underscoring its therapeutic promise.

## Data Availability

The original contributions presented in the study are included in the article/**Supplementary Material**. Further inquiries can be directed to the corresponding author.
