# Supplementary material for: Diethyldithiocarbamate-copper complex ignites the tumor microenvironment through NKG2D-NKG2DL axis
Source: Front Immunol. 2025 Feb 12;16:1491450. doi: 10.3389/fimmu.2025.1491450 (PMC11860975; doi:10.3389/fimmu.2025.1491450)
Supplement: Supplementary file 1 [file DataSheet1.docx]

***Supplementary Material***

**Diethyldithiocarbamate-copper complex ignites the tumor microenvironment through NKG2D-NKG2DL axis**

Daciana C. Dumut^1,2^; Marian Hajduch^3,4^; Amanda M. Zacharias^5^; Qingling Duan^5,6^; Ivo Frydrych^3^; Zuzana Rozankova^3,4^; Miroslav Popper^3,4^; Dušan Garić^7^; Radu Alexandru Paun^2,8^; Amanda Centorame^1,2^; Juhi Shah^2^; Martin Mistrik^3,4^; Petr Dzubak^3,4^; Juan B. De Sanctis^3,4^; Danuta Radzioch^1,2,3,4,^*

1. Department of Experimental Medicine, Faculty of Medicine, McGill University, Montreal, Qc. Canada;
2. The Research Institute of the McGill University Health Centre, Infectious Diseases in Global Health Program, Montreal, Qc. Canada;
3. Institute of Molecular and Translational Medicine, Faculty of Medicine and Dentistry, Palacky University, Olomouc, Czech Republic;
4. Czech Advanced Technology and Research Institute, Palacky University Olomouc, Krizkovskeho 511/8, 77900 Olomouc, Czech Republic;
5. Department of Biomedical & Molecular Sciences, Faculty of Health Sciences, Queen’s University, Kingston, ON, Canada;
6. School of Computing, Department of Biomedical & Molecular Sciences, Queen’s University, Kingston, ON, Canada;
7. Department of Developmental Neurobiology, St. Jude Children's Research Hospital, Memphis, TN 38105, USA;
8. Department of Biomedical Engineering, McGill University, Montreal, Qc, Canada.

Keywords: Copper bis-diethyldithiocarbamate/ disulfiram/ colorectal cancer/ NK cells/ NKG2D

**Supplementary Material**

**Supplementary Methods**

*Cell Culture*

CT-26 cells were grown in Roswell Park Memorial Institute 1640 (RPMI). The human CRC cell lines HT-29 (ATCC HTB-38), HCT116 parental (KRAS^G13D^/KRAS^WT^, ATCC CCL-247 via Horizon cat. HD PAR-007), HCT116 WT (KRAS ^WT^/KRAS ^KO^, cat. HD-104-008), and HCT116 KRAS^G13D^ (KRAS^G13D^/ KRAS ^KO^, cat. HD 104-011) were maintained in McCoy's 5A medium. All were supplemented with 10% (v/v) foetal bovine serum (FBS), 1% (v/v) penicillin/streptomycin, and maintained in a humidified atmosphere of 5% CO_2_ at 37°C. All cell culture reagents were purchased from Wisent.

*Clonogenic Assay*

The cells were seeded at 300 cells/well in 6-well plates, treated with CuET in DMSO every second day for seven days, and incubated at 37⁰C with 5% CO_2._ On day 7, cells were washed and stained with 0.5% (w/v) crystal violet (Sigma-Aldrich) in 70% ethanol. The total number of colonies was then manually counted. Experiments were done in triplicates.

*Migration-Invasion Assay*

Cells were serum-starved for 24h before seeding. Cells were detached with 0.05% trypsin-EDTA and seeded in FBS-free media at 3 × 10^5^ cells per well in 12-well 8.0μm pore-size Boyden chamber inserts (ThermoFisher) pre-coated with rat tail collagen I (Gibco). In the bottom compartment, CuET was introduced at various concentrations in the media supplemented with FBS. Cells were incubated at 37⁰C with 5% CO_2_ for 24h. Boyden chamber inserts were fixed with 5% (v/v) glutaraldehyde (Sigma-Aldrich), washed, and stained with 0.5% (w/v) crystal violet. The interior of the chambers was cleaned to remove non-migratory cells using cotton swabs. Four fields per insert were photographed with an Olympus IX51 inverted microscope with a 20X objective and migratory cells were counted in ImageJ Fiji with the Cell Counter plugin.

*Western blotting*

Cells were seeded in 60mm dishes at 8 × 10^5^ cells per dish. On the second day, the cells were treated with 0µM, 1, 2, or 5 µM CuET in DMSO for a duration of 24h. Cells were lysed with radioimmunoprecipitation assay (RIPA) buffer supplemented with a protease inhibitor mini tablet (Roche). The lysates were cleared by centrifugation at 14,000  rpm for 15 min. Protein concentrations were measured using a Pierce BCA assay kit (ThermoFisher). Equal amounts of protein lysates (20 µg) were separated using a 4–20% Mini-PROTEAN® TGX™ Precast Protein Gel, 15-well, 15 µl #4561096 (Bio-Rad) sodium dodecyl sulfate-polyacrylamide gel electrophoresis (SDS-PAGE) and transferred to PVDF membranes (Bio-Rad). Blots were incubated with i) primary antibodies against β-actin (MAB1501R, Millipore, 1:5000) paired with secondary HRP antibody (#405306, BioLegend, 1:10000), ii) with primary antibodies anti-PARP (#9542S, 1:1000) and iii) anti-XIAP (#2045S, 1:1000) antibodies (Cell Signaling Technologies) paired with secondary HRP antibody (#31458, Invitrogen, 1:10000). The blots were developed using Bio-Rad ECL solution and imaged using the Bio-Rad ChemiDoc MP Imaging System.

*Blood biochemistry and hematology*

Mice were treated with CuET or vehicle as per the schedule in Fig.2 A, and received the last dose of of treatment i.p. 24h before the blood harvest. Blood was collected and separated into two fractions: 1) whole blood in 10uL of 0.5M EDTA, pH 8.0, (Invitrogen) for cellular blood count analysis and 2) in Z-Gel clot activator microtubes (Sarstedt) from which serum was isolated for biochemical analysis. Blood count was performed by Heska ELEMENT HT5 automated cell counting technology which employs three measurement methods: Electrical Impedance for red blood cells and platelets, Colorimetric for hemoglobin, and Flow Cytometry by Laser for white blood cell data (size and density). The blood biochemistry analyses were performed by Beckman UniCell DXC600 employing photometry, potentiometry, and ion-selective electrode methods to analyze blood biochemistry. Biochemical and haematological analyses were performed by Biovet®, Saint-Hyacinthe, QC, Canada.

*TUNEL Assay*

The assay was performed according to the manufacturer's instructions using an *in-situ* apoptosis detection kit (MK500, TakaraBio) to detect DNA degradation in the tumors of BALB/c mice treated with vehicle or albumin-CuET. Briefly, slides were de-paraffinized and hydrated in decreasing concentrations of ethanol. The slides were washed in PBS and incubated for 15 min with proteinase K+ (20 μg/ml). Endogenous peroxidases were inactivated by applying 0.3% H_2_O_2_ at room temperature for 15 - 30 min. TUNEL reaction mixture was added to the sections for end-labelling of DNA with fluorescein-dUTP, and slides were incubated in a humidified chamber in the dark at 37°C for 1 h. Anti-FITC HRP conjugate was added and incubated at 37°C for 30min. Next, the slides were washed and DAB substrate was added for 15 min at room temperature. Finally, the slides were washed and counterstained using a solution of 1.5% methyl green (Sigma). Scanning was performed using a Leica Aperio AT Turbo digital pathology scanner at 40X magnification and 25 microns/pixel.

*Mouse NK, NKT Cytotoxicity Assays*

500 MC-38 cells (Target) per well were seeded into 96-well plates with 2.5µM/ mL of IncuCyte Cytotox Green Reagent (Essen Bioscience, 4633) 24h before co-culture with murine hepatocytes (Effector) from mice previously treated with either CuET or vehicle, at the Effector/Target ratio of 50:1. When using FACS-sorted and enriched NK or NKT cells Effector/Target ratio of 5:1 was used. Plates were incubated in Sartorius IncuCyteS3 for 24h using a green fluorescence/phase module to monitor green, fluorescent objects every two hours. MFI/well adjusted to time point 0 was acquired, representing the target cell death.

*Human PBMC isolation, NK and T cell enrichment*

Human PBMC were isolated from fresh blood buffy coats using a standard Ficoll-Hypaque assay. NK and T cells were further separated from PBMC by negative selection using Mojo Sort NK and Mojo Sort T cells (BioLegend, cat. 480053, and 480022). The purified NK cells were 95.1 ± 2.3% for CD56 expression (clone HCD56, BioLegend), and the purified CD3 cells were 98.4 ± 0.8% (clone OKT3, BioLegend). The expression of CD8 cells (clone HIT8a, BioLegend) was 42.8 ± 4.9% in NK cells and 38.9 ± 7.8% in T cells.

*Generation of antibody Fab fractions*

To generate antibody Fab fractions for anti-ligand treatment, MICA (clone 159227, R&D Systems), ULBP1 (clone 170818, R&D Systems), and ULBP2/5/6 (clone 165903, R&D Systems) antibodies underwent pepsin treatment using the Thermo Scientific™ Pierce™ F(ab')2 Preparation Kit (cat.44988), optimized for human, rabbit, and mouse IgG. Pepsin digestion effectively produced IgG2 fractions. In the NK cell functional assay against HCT116 and HT-29 (target cells) stimulated with 1nM CuET for 18h, 1 million target cells were incubated with 0, 1, 10, 20, and 50 ng Fab fractions of MICA, ULBP1, and ULBP2 antibodies for 1 hr at 4°C. Subsequently, HCT116 and HT29 cells were set to 50000 cells and challenged with different effector cell concentrations at Effector:Target ratios of 40:1, 20:1, 10:5, and 5:1. A set of three samples without lymphocytes was used as control for the assay.

**
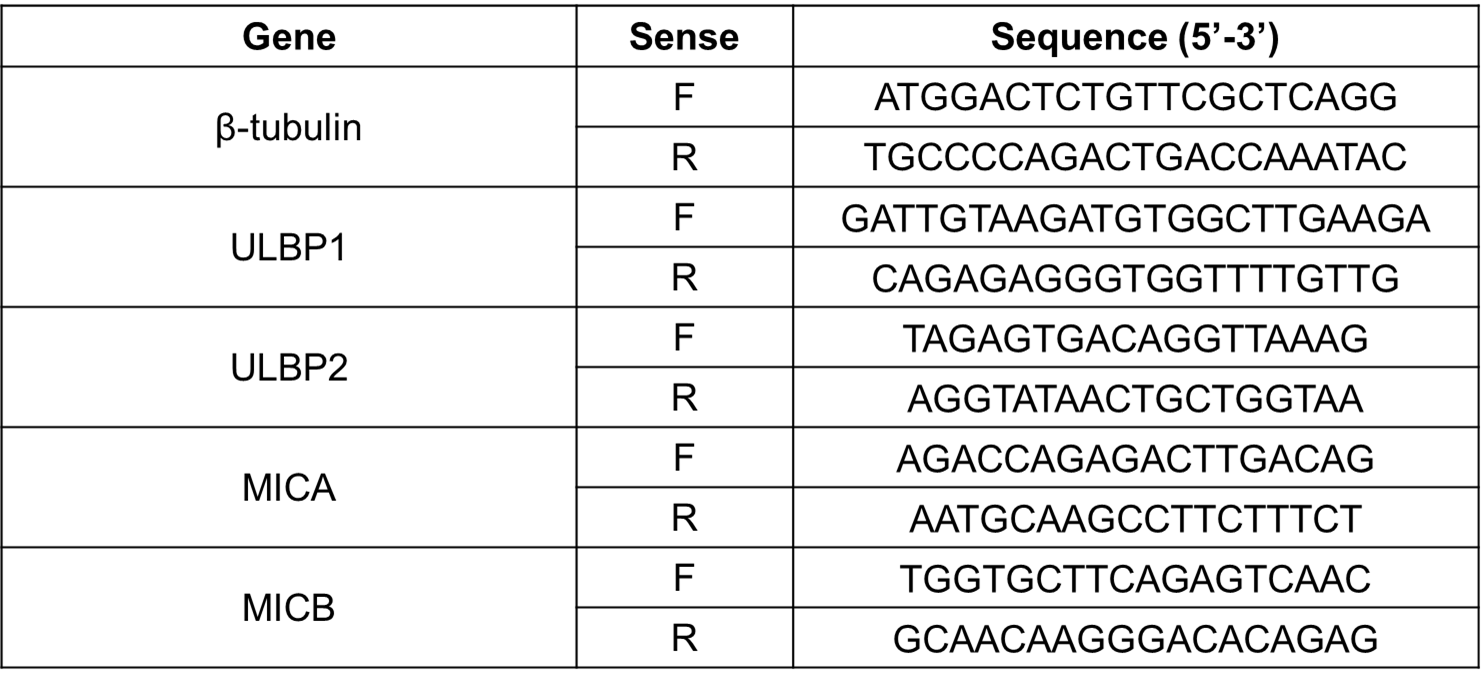
Sup. Table 1. Primer sequences for qPCR analysis of mRNA expression in HCT116 and HCT116 G13D cells.**

**Sup. Table 2.** **Biochemical parameters in BALB/c mice systemically treated with vehicle or CuET remain stable.** Four injections of albumin-CuET nanoparticles over 17 days did not alter the blood biochemical parameters in BALB/c mice of mixed sexes treated with vehicle or CuET as described in Materials and Methods. Twenty-four hours after the last treatment, blood was taken and serum was separated. Twenty-four biochemical parameters were analysed.


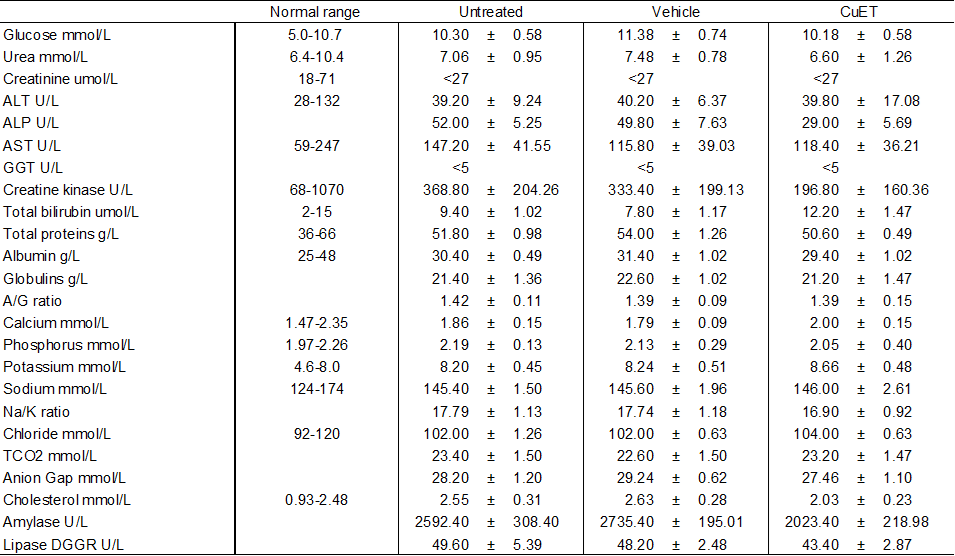


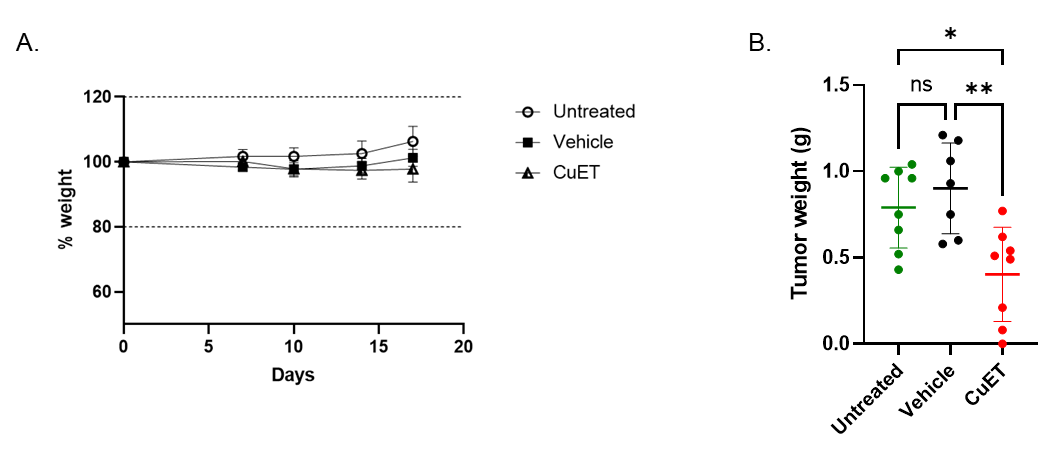


**Sup. Figure 1. A) The weight of BALB/c mice was not affected by CuET treatment. B) The CT-26 tumor weight is significantly reduced by CuET treatment.** A) BALB/c mice were treated with four doses of i.p. albumin-CuET nanoparticles or vehicle over the course of 17 days, and mouse weight was monitored daily. No statistically significant or biologically significant (indicated by dotted lines) weight change was observed when compared to untreated animals. Two-way ANOVA with Tukey’s correction where n= 5 mice per group, **p<0.05, **p<0.005, ***p<0.0005, and ****p<0.0001.* B) Tumor weight at the endpoint was significantly reduced by CuET treatment (n= 8) as compared to untreated (n=8) and vehicle (n=7) groups. Data expressed as mean ± SD. Welch ANOVA with Dunnett’s T3 correction where **p<0.05, **p<0.005, ***p<0.0005 and ****p<0.0001*.

**
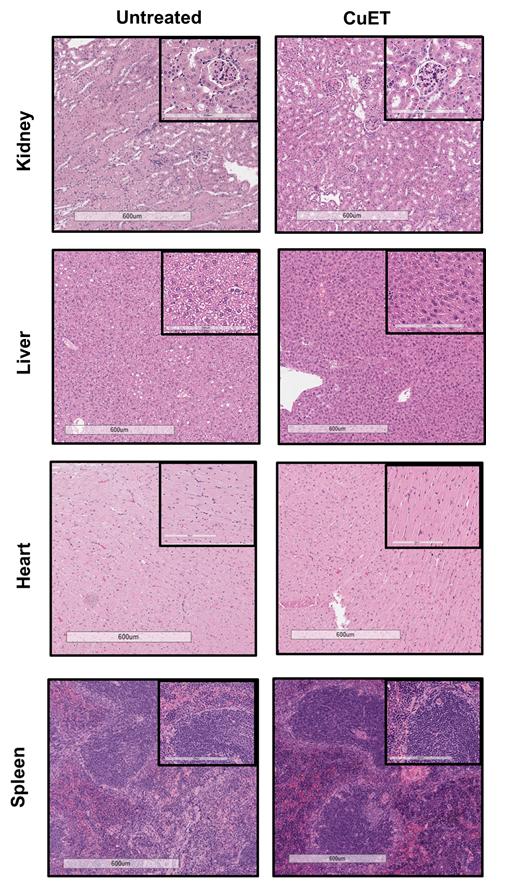
Sup. Figure 2. Hematoxylin and eosin staining of internal organs of BALB/c mice showed no signs of internal organ toxicity induced by CuET treatment.** BALB/c mice were treated with four doses of i.p. albumin-CuET nanoparticles over seventeen days. Twenty-four hours after the last treatment, the internal organs were harvested, fixed, and prepared for histological analysis. Images show 4X and 20X (inlet). H&E staining showed no morphological changes, such as hypertrophy of the kidney, liver, heart, and spleen tissue in the CuET-treated animals compared to untreated animals.

*
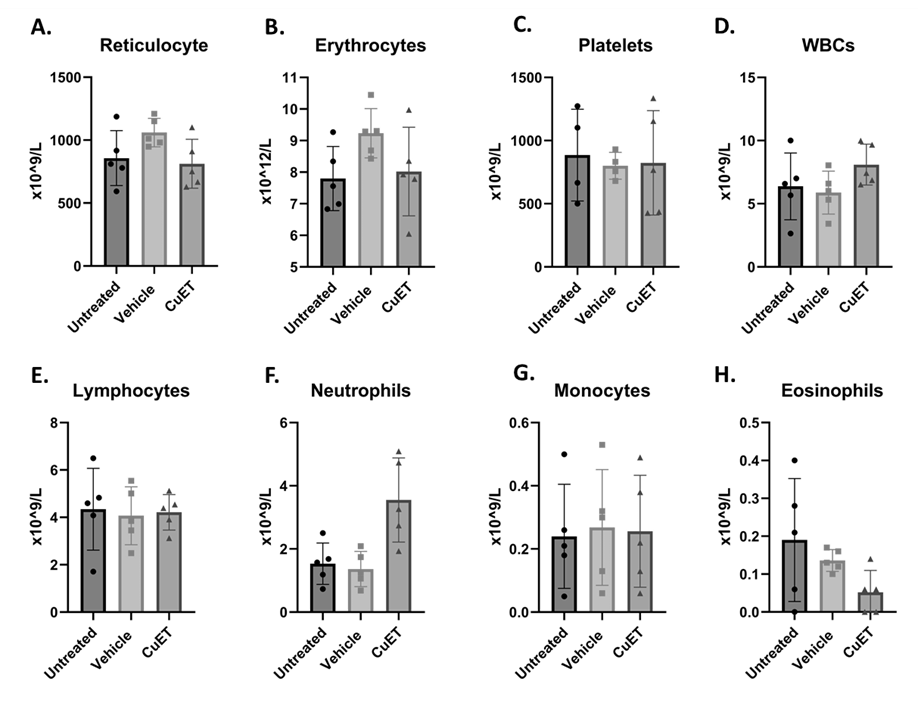
*

**Sup. Figure 3. Cellular blood counts remained stable in CuET-treated mice compared to vehicle and untreated controls.** Four injections of albumin-CuET nanoparticles did not affect the blood cell counts in BALB/c mice of mixed sexes treated with vehicle or CuET. Twenty-four hours after the last treatment, blood was taken and cell counts were performed. No statistically significant change was observed between any groups with Welch’s ANOVA, n= 5 per group.


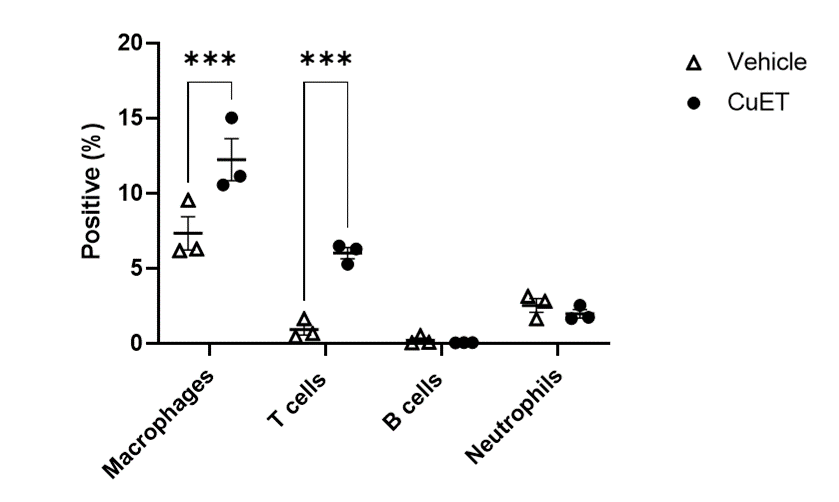


**Sup. Figure 4. Immunohistochemical staining of CT-26 tumors from mice treated with CuET show a significantly increased infiltration of macrophages and T cells.** Quantification of infiltration of lymphocytes in tumor sections from vehicle-treated and CuET-treated mice. Data expressed as mean ± SEM. Sidak’s Two-Way ANOVA, where **p<0.05, **p<0.005, ***p<0.0005 and ****p<0.0001* for % positive cells per field with 10 image fields analyzed for each mouse tumor, n=3 per group.


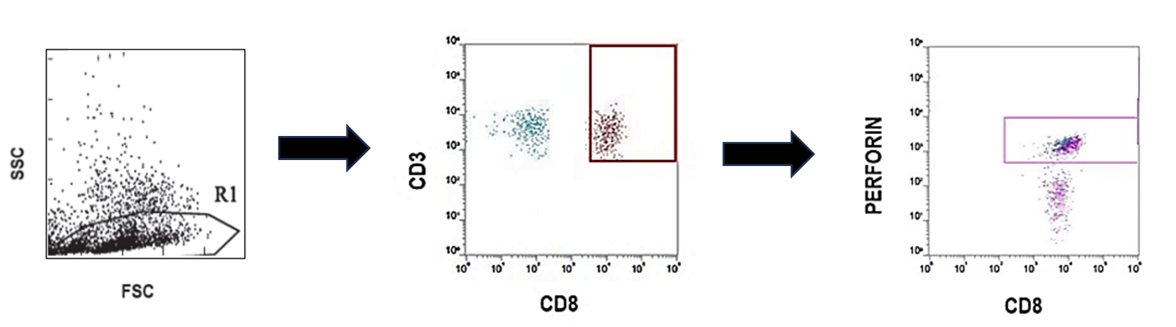


**Sup. Figure 5: Gating strategy on CD8+ cells for perforin expression analysis in control and CuET treated cells.** Perforin expression is increased by CuET stimulation of C57BL/6 mouse splenocytes ex-vivo for 18h (Main manuscript Figure 3. J-K).


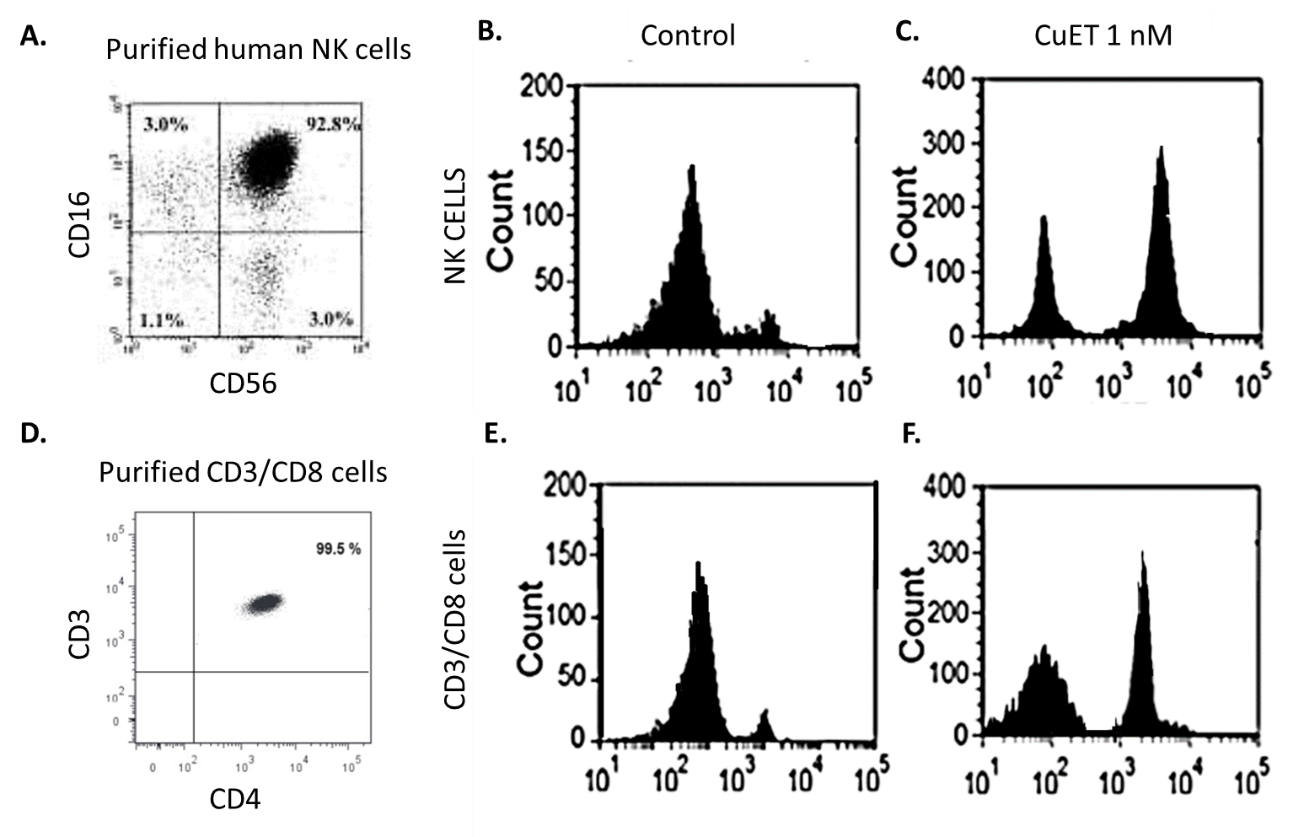


**Sup. Figure 6: Flow cytometry plots showing purification of NK and T cells after negative selection with MojoSort and NKG2D expression in control and CuET treated cells.** A, D) Populations of purified NK and T cells. B, C) NKG2D positive count in NK cells in control and after 18h of 1nM CuET stimulation. E, F) NKG2D positive count in T cells in control and after 18h of 1nM CuET stimulation.


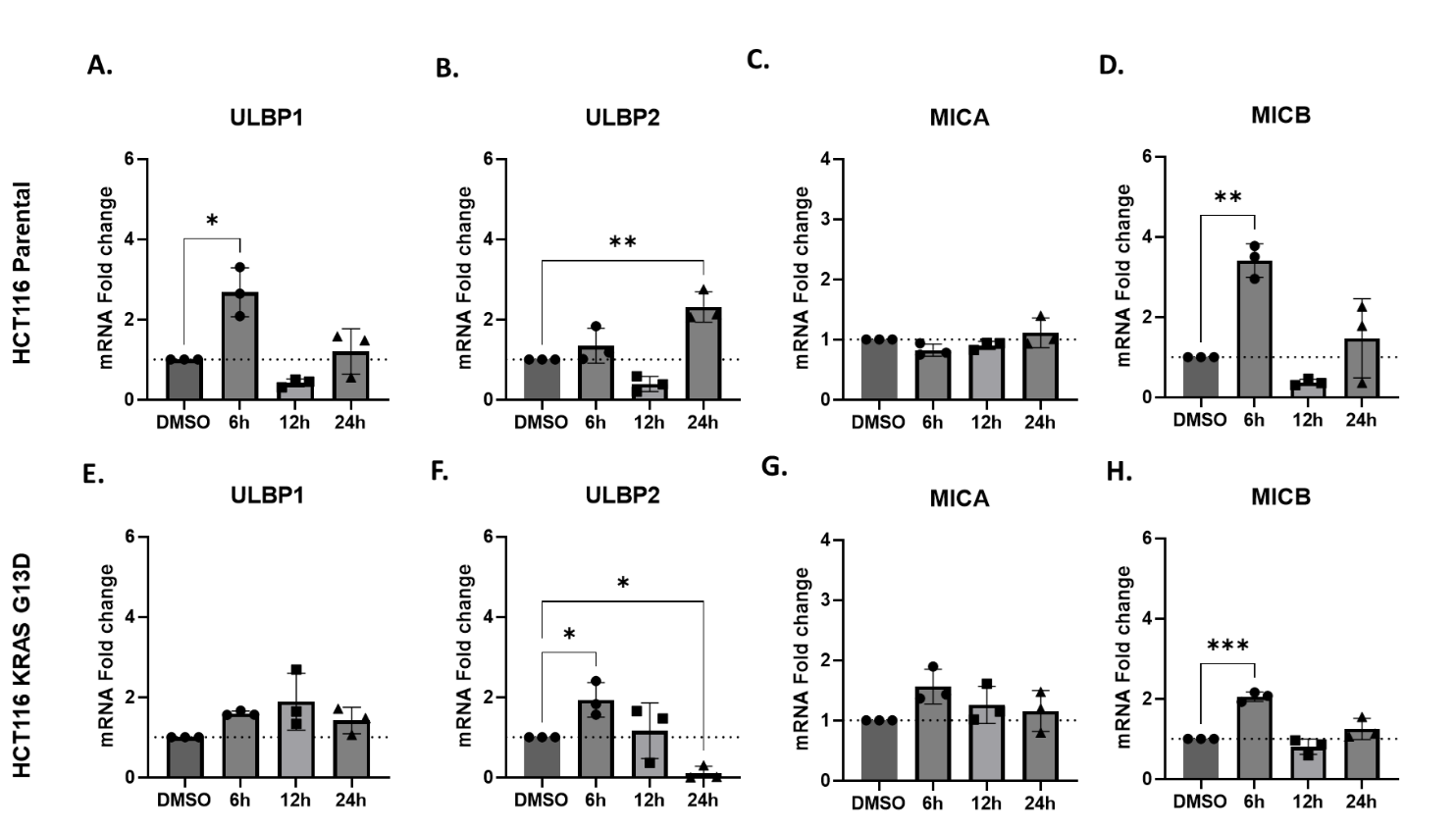


**Sup. Figure 7: CuET induces mRNA expression of NKG2D receptor ligands in HCT116 cells.** A, B, C, D) ULBP1, ULBP2, MICA, MICB gene modulation at 6h, 12h, and 24h post-treatment with 1µM CuET, in HCT116 parental cells. E, F, G, H) ULBP1, ULBP2, MICA, MICB gene modulation at 6h, 12h, and 24h post-treatment with 1µM CuET, in HCT116 G13D cells. (n = 3). Data represented as means ± SD. Dunnett’s Two-Way ANOVA, where **p<0.05, **p<0.005, ***p<0.0005 and ****p<0.0001.*

*
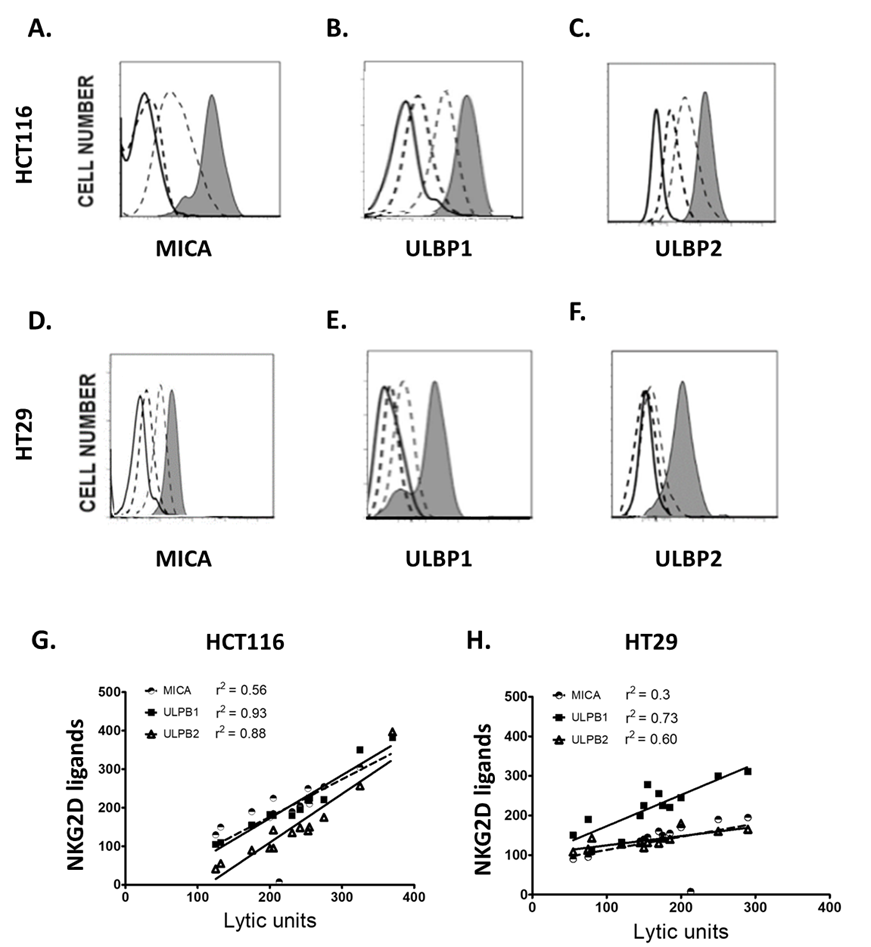
*

**Sup. Figure 8: Antibody-blocking of NKG2D ligands on HCT116 and HT29 CRC cells ablates cytotoxic activity of NK cells.** A, B, C) Efficacy of antibody-blocking of NKG2D ligands on HCT116 and on D, E, F) HT29 cell lines treated with 1 nM CuET, incubated with the antibody against MICA, ULBP1, and ULBP2 Fab fractions. In gray the expression without blocking each respective antibody. The continuous line is the background. The two dotted lines, black and grey, correspond to 10 ng of the antibody and 20 ng of the antibody, respectively. G, H) Correlation analysis between lytic activity of NK cells against HCT116 and HT29 cells and expression of NKG2D ligands MICA, ULBP1, and ULBP2. Effector to target ratios analysed are 40:1, 20:1, 10:5 and 5:1. The number of HCT116 and HT29 treated cells was set to 50000 cells. A set of three samples without lymphocytes was used as control of the assay.

**
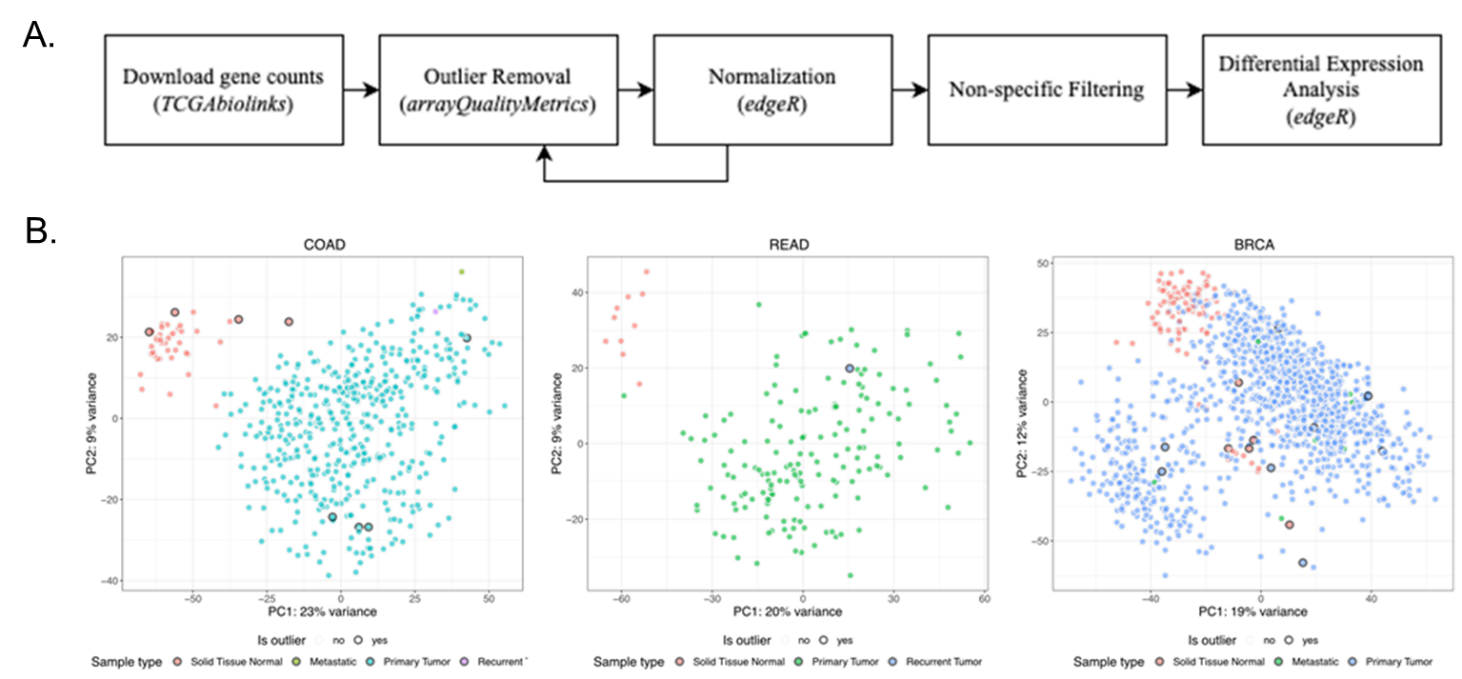

Sup. Figure 9: Pipeline used and outlier exclusion for differential gene expression of NKGD2 cancer ligands in tumor samples versus normal tissue from projects TCGA-COAD (colon adenocarcinoma), -READ (rectal adenocarcinoma), and –BRCA (breast carcinoma).** A) Flowchart of differential expression analysis methods. Gene expression data from The Cancer Genome Atlas Program projects TCGA-READ, TCGA-COAD, and TCGA-BRCA were downloaded using TCGAbiolinks. Outlier samples were detected using arrayQualityMetrics before and after gene count normalization with edgeR. Highly variable genes were then selected based on their median absolute deviation. Finally, differential expression analyses of 11 candidate genes were performed with edgeR. B) Principal component analysis highlighting outlier samples and clustering by sample type. Variance stabilizing transformation and principal component analysis were performed with DESeq2. Data points are coloured by sample type and outlined in black if considered outliers. Using arrayQualityMetrics, samples were considered outliers if they were either marked an outlier before and after normalization or marked as an outlier by multiple metrics after normalization. COAD dataset (n=8), READ dataset (n=1), and BRCA dataset (n=13).

**
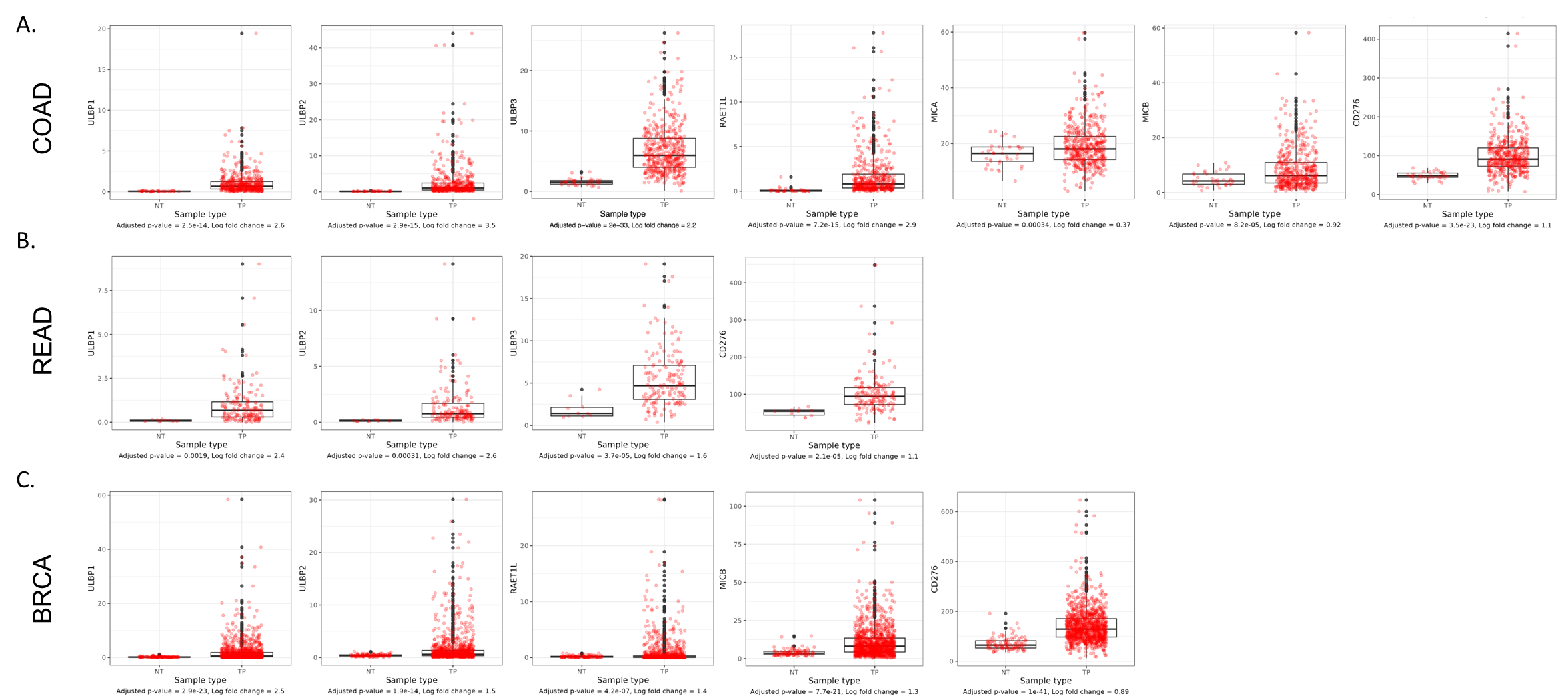
**

**Sup. Figure 10: Volcano plots of the comparative analysis of NKG2DL expression in tumor samples (TP) versus normal tissue (NT) across three TCGA projects.** A) COAD (colon cancer) dataset, n=504 samples. B) READ (rectal cancer) dataset, n=176 samples. C) BRCA (breast cancer), n=1206 samples. The following 11 genes were analyzed: ULBP1, ULBP2, ULBP3, MICA, MICB, CD274, RAET1L, RAET1G, HCST, CD276, and KLRK1.

**Appendix**

**Source File Fig.1G.**


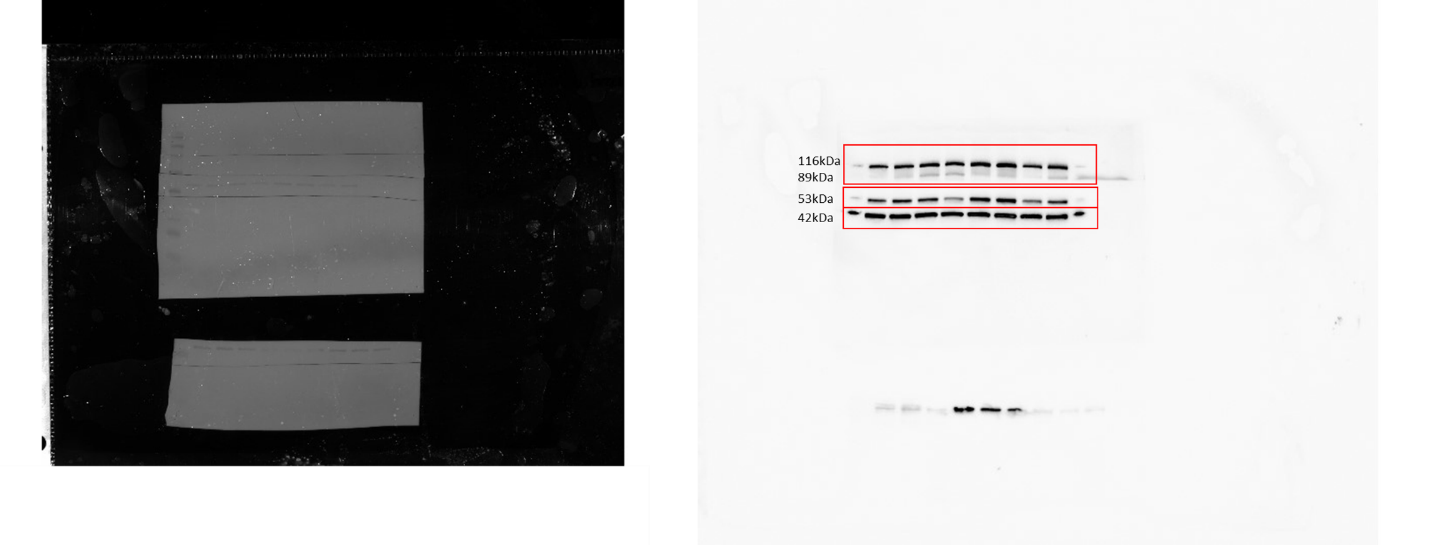


**Original immunohistology images (higher resolution)**


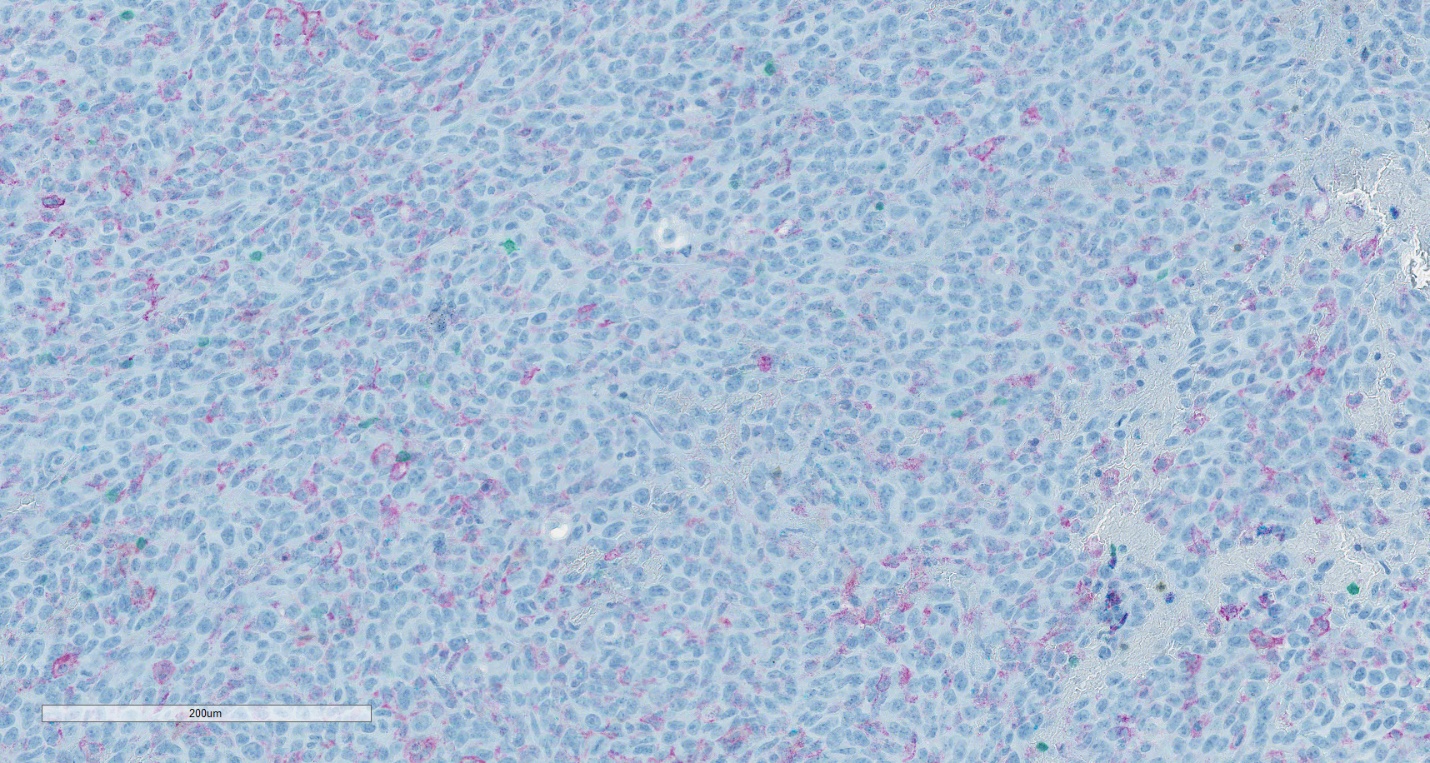
**CT-26 tumor, vehicle-treated mouse (Fig. 2 M)**


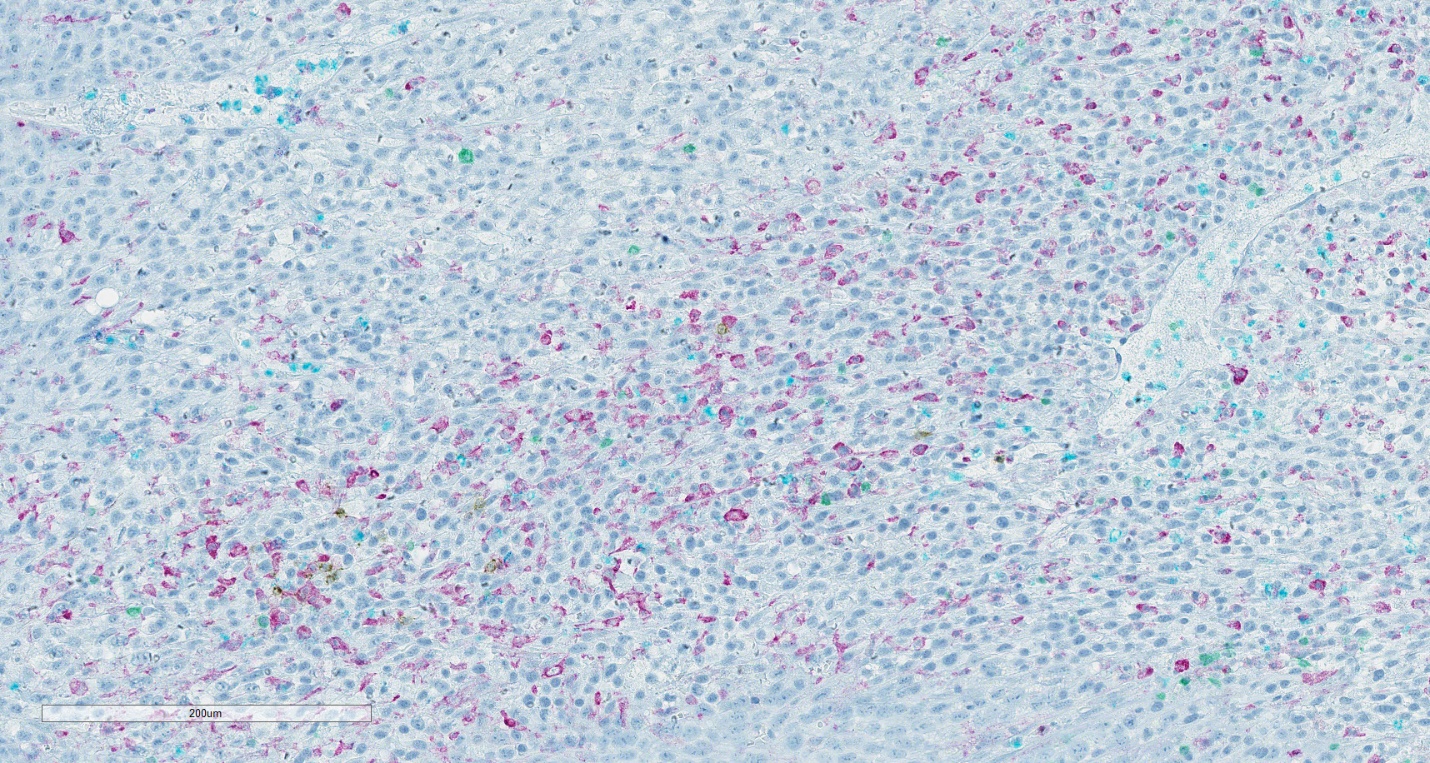
**CT-26 tumor, CuET-treated mouse (Fig.2 N)**


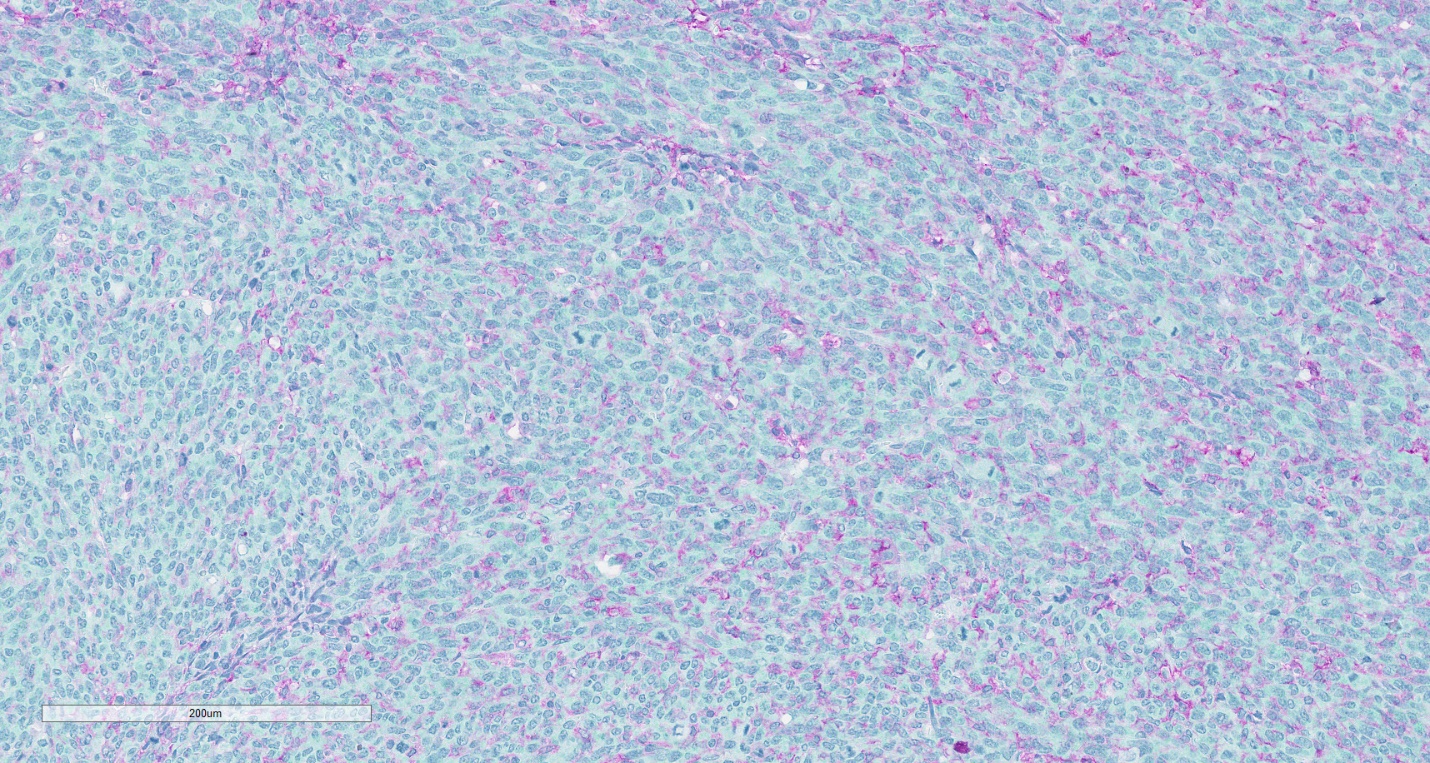
**MC-38 tumor, vehicle-treated mouse (Fig.3 M)**


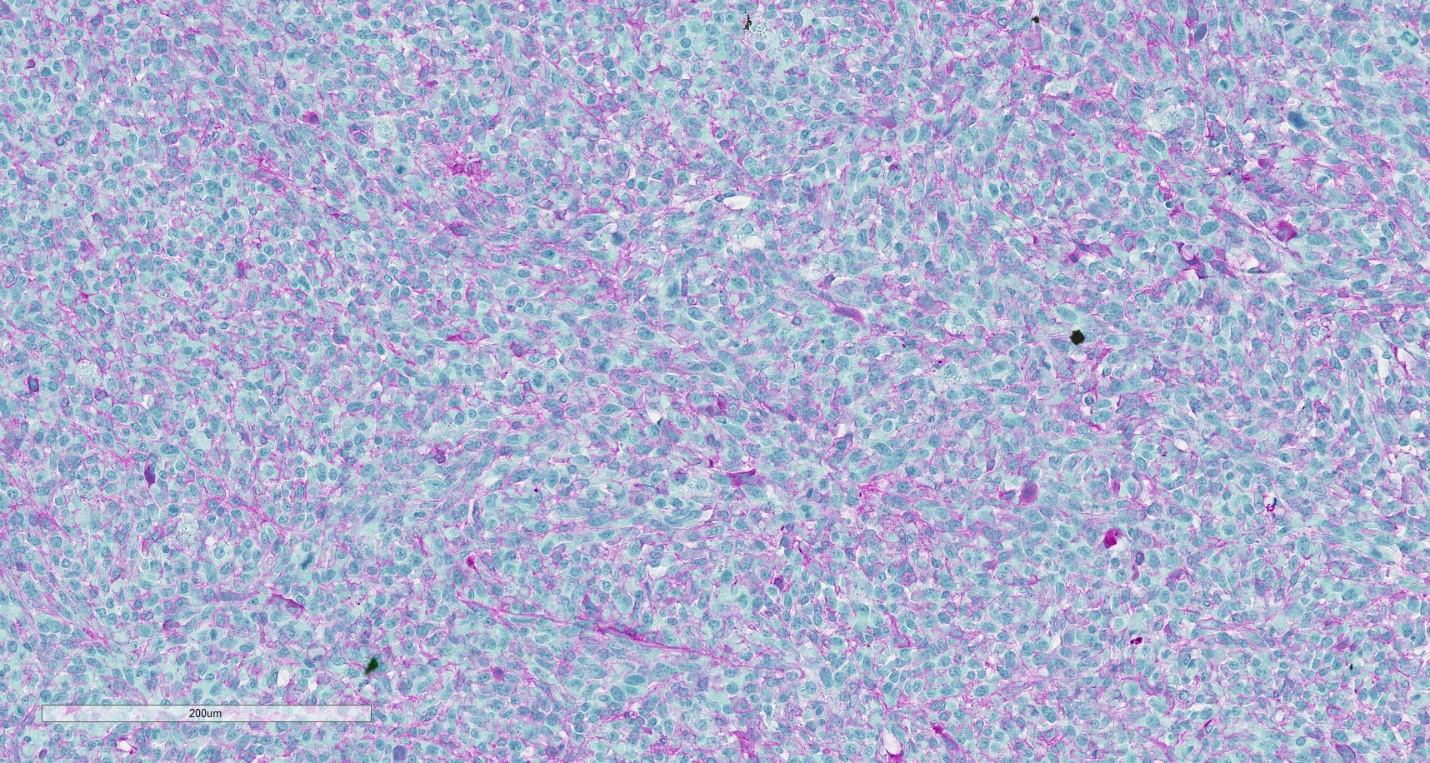
**MC-38 tumor, CuET-treated mouse (Fig.3 N)**


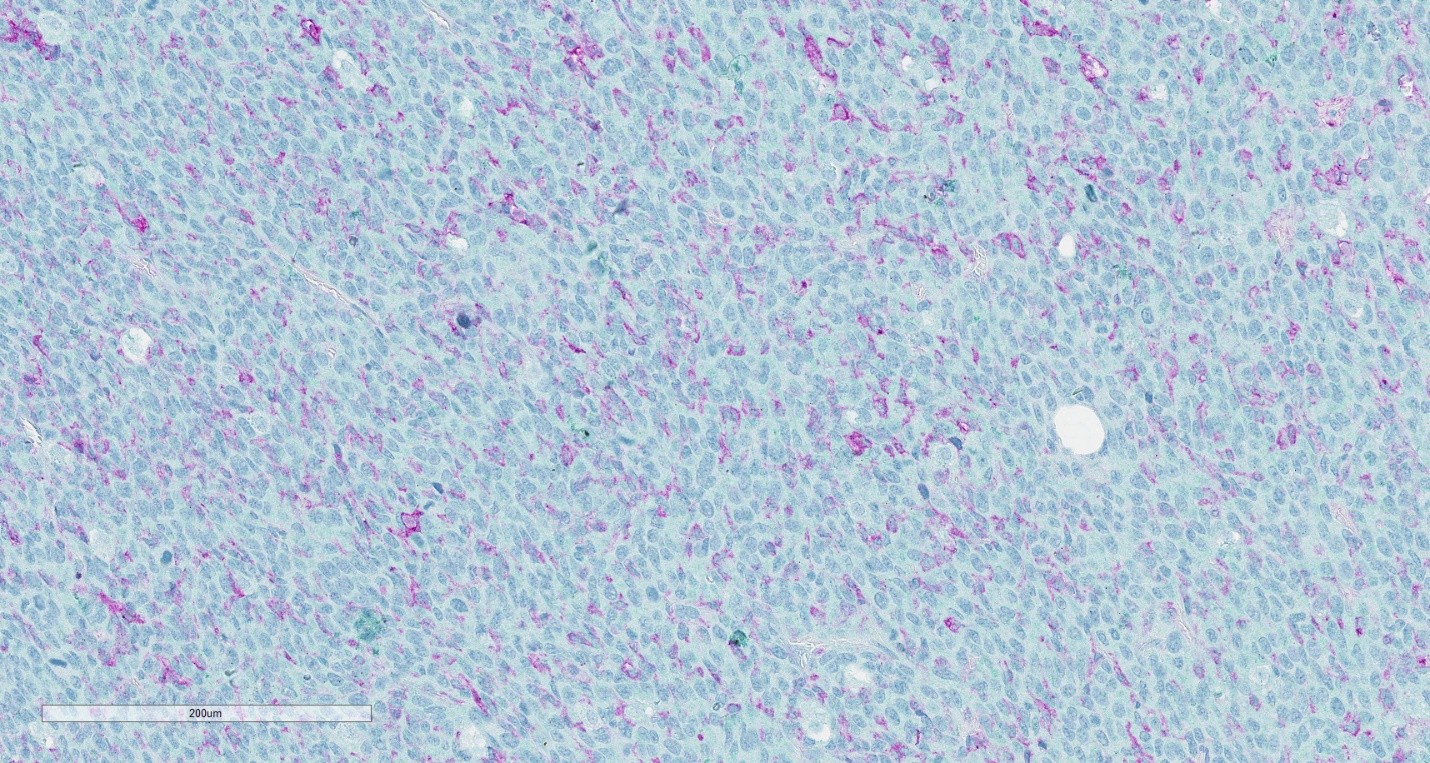
**CT-26 tumor, vehicle-treated mouse**


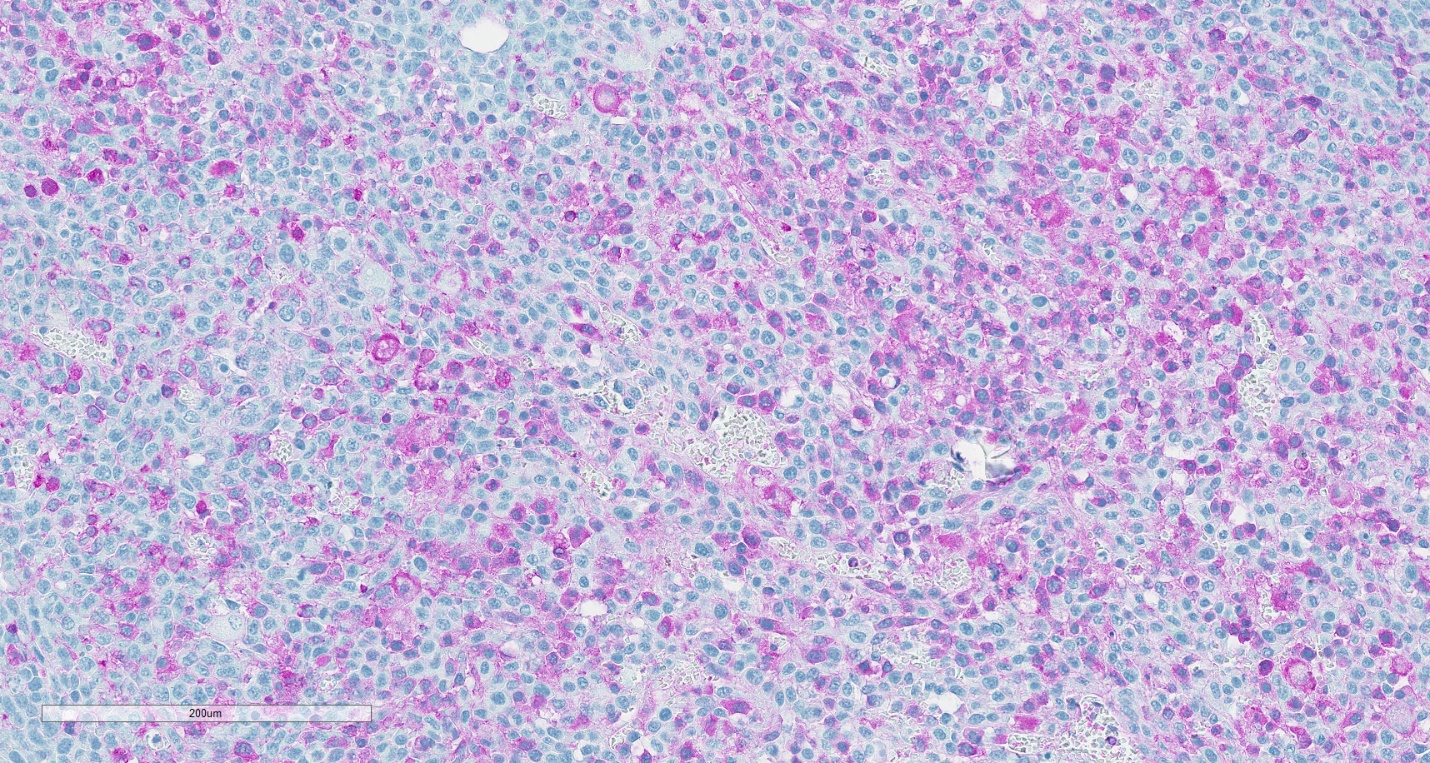
**CT-26 tumor, CuET-treated mouse**
